# TRPA1 mediates damage of the retina induced by ischemia and reperfusion in mice

**DOI:** 10.1038/s41419-020-02863-6

**Published:** 2020-08-15

**Authors:** Daniel Souza Monteiro de Araújo, Francesco De Logu, Chiara Adembri, Stanislao Rizzo, Malvin N. Janal, Lorenzo Landini, Alberto Magi, Gianluca Mattei, Nicoletta Cini, Pablo Pandolfo, Pierangelo Geppetti, Romina Nassini, Karin da Costa Calaza

**Affiliations:** 1 Department of Neurobiology and Program of Neurosciences, Institute of Biology, Fluminense Federal University, Niterói, Brazil; 2 Department of Health Sciences, Section of Clinical Pharmacology and Oncology, University of Florence, Florence, Italy; 3 Department of Neurosciences, Psychology, Drug Research and Child Health (NeuroFarBa), Division of Ophthalmology, University of Florence, Florence, Italy; 4 Department of Epidemiology and Health Promotion, New York University College of Dentistry, New York, NY USA; 5 Department of Information Engineering, University of Florence, Florence, Italy; 6 General Laboratory, Careggi University Hospital, Florence, Italy

**Keywords:** Pharmacology, Retina, Preclinical research

## Abstract

Oxidative stress is implicated in retinal cell injury associated with glaucoma and other retinal diseases. However, the mechanism by which oxidative stress leads to retinal damage is not completely understood. Transient receptor potential ankyrin 1 (TRPA1) is a redox-sensitive channel that, by amplifying the oxidative stress signal, promotes inflammation and tissue injury. Here, we investigated the role of TRPA1 in retinal damage evoked by ischemia (1 hour) and reperfusion (I/R) in mice. In wild-type mice, retinal cell numbers and thickness were reduced at both day-2 and day-7 after I/R. By contrast, mice with genetic deletion of TRPA1 were protected from the damage seen in their wild-type littermates. Daily instillation of eye drops containing two different TRPA1 antagonists, an oxidative stress scavenger, or a NADPH oxidase-1 inhibitor also protected the retinas of C57BL/6J mice exposed to I/R. Mice with genetic deletion of the proinflammatory TRP channels, vanilloid 1 (TRPV1) or vanilloid 4 (TRPV4), were not protected from I/R damage. Surprisingly, genetic deletion or pharmacological blockade of TRPA1 also attenuated the increase in the number of infiltrating macrophages and in the levels of the oxidative stress biomarker, 4-hydroxynonenal, and of the apoptosis biomarker, active caspase-3, evoked by I/R. These findings suggest that TRPA1 mediates the oxidative stress burden and inflammation that result in murine retinal cell death. We also found that TRPA1 (both mRNA and protein) is expressed by human retinal cells. Thus, it is possible that inhibition of a TRPA1-dependent pathway could also attenuate glaucoma-related retinal damage.

## Introduction

Glaucoma is the most common optic neuropathy potentially causing blindness worldwide^1^. Increased intraocular pressure (IOP), the major risk factor for glaucoma and the object of therapeutic intervention in glaucoma patients, can elicit retinal ischemia and the loss of retinal ganglion cells^2^, reduced visual acuity and eventually, blindness^1–3^. Despite considerable efforts directed toward identifying the cellular and molecular changes that may lead to retinal cell death^4^, the major contributing pathways are not yet understood. The ischemic insult has been reported to originate from an initial calcium elevation and ensuing stimulation of excitotoxic mechanisms mediated by activation of purinergic P2X7 or glutamate receptors of the AMPA/NMDA subtype^3,5,6^. In a commonly used model of glaucoma, ischemia, and reperfusion (I/R) in rodent retina^7^ is associated with increased reactive oxygen species (ROS) levels, tissue damage and cell death^3^. Increased oxidant levels have been proposed as the causal factor of glaucomatous retinal ganglion cell loss^8–11^, an explanation that is supported by data showing that administration of antioxidants protects retinal cells from injury following retinal I/R^12–15^. However, the specific pathways linking the oxidative stress burden to retinal damage remain unspecified.

Transient receptor potential (TRP) channels are expressed by a large variety of cells in several tissues and organs^16–18^. Some TRPs are activated by changes in the redox state of the milieu with pronounced differences in their sensitivity^19,20^. TRP ankyrin 1 (TRPA1) is markedly sensitive to the oxidative burden of the tissue^19^, and is efficiently activated by an unprecedented series of oxidative, nitrative and carbonyl species (ROS, RNS, and RCS, respectively)^21–24^. The ability of oxidative stress byproducts to gate TRPA1 has been identified as a major mechanism in models of inflammatory^25,26^ and neuropathic pain^27,28^. Notably, in a model of sciatic nerve injury, Schwann cell TRPA1 was found to sense the macrophage-dependent oxidative stress burst, sustaining ROS and RCS production and pain signals *via* an NADPH oxidase 1 (NOX1)-mediated pathway^28^. Recently, we reported that the TRPA1 protein was present in the chick retina^29^, and that pharmacological blockade of this channel attenuated the increase in lactate dehydrogenase, a biomarker of tissue damage evoked by oxygen and glucose deprivation^29^. These findings suggest that TRPA1 may be implicated in retinal injury, but several issues remain to be investigated, including whether TRPA1 is present in the rodent and human retina, and how it participates in the damage cascade evoked by I/R.

Here, we report that TRPA1 is widely expressed by Müller, horizontal, amacrine, and ganglion cells of both mouse and human retina. We also show in a mouse model of retinal damage that genetic deletion or pharmacological blockade of TRPA1 attenuated the increase in the apoptosis biomarker, active caspase-3, reduced retinal cell death and preserved retinal tissue thickness. Antioxidant treatment provided similar protections, indicating that both oxidative stress and TRPA1 are entirely responsible for the tissue damage. Finally, and surprisingly, we found that genetic and pharmacological inhibition of TRPA1 attenuated macrophage accumulation and the increase in the oxidative stress end-product, 4-hydroxynonenal (4-HNE). This suggests that TRPA1 expressed by one or more retinal cells elicits the oxidative stress burden that, in an autocrine or paracrine manner, facilitates the retinal damage caused by I/R.

## Materials and methods

### Animals

C57BL/6J mice (male, 20–30 g, 6–8 weeks; Envigo, Milan, Italy), littermate wild-type (*Trpa1*^+/+^) and TRPA1-deficient (*Trpa1*^*−/−*^) mice (male, 20–30 g, 6–8 weeks), generated by heterozygotes on a C57BL/6J background (B6.129P-*Trpa1*^tm1Kykw^/J; Jackson Lab, Bar Harbor, ME, USA)^30^, wild type (*Trpv4*^+/+^) and TRPV4-deficient (*Trpv4*^*−/−*^) mice (male, 20–30 g, 6–8 weeks) generated by heterozygotes on a C57BL/6 background^31^, TRPV1-deficient mice (*Trpv1*^*−/−*^; B6.129×1-*Trpv1*^tm1Jul^/J) backcrossed with C57BL/6J mice (*Trpv1*^*+/+*^) (Jackson Lab, Bar Harbor, ME, USA; 25–30 g, 5–8 weeks) and *Advillin-Cre* mice expressing a Cre-recombinase from the locus of the primary sensory neuron-specific gene *Advillin*^32^ backcrossed with 129S-Trpa1^tm2Kykw^/J mice (*floxed TRPA1,Trpa1*^*fl*^*/*^*fl*^, Stock No: 008649; Jackson Lab, Bar Harbor, ME, USA) *(Adv-Cre*;*Trpa1*^*fl/fl*^) resulting in a selective TRPA1 mRNA attenuation in nociceptors, were used. The total number of mice used was 114. The group size for each experiment was determined by sample size estimation^33^ to obtain a desired power, on the basis of both our past experience in similar experimental settings and data published by others. No animals were excluded from the experiments.

Allocation concealment of mice to vehicle(s) or treatment(s) group was performed using a randomization procedure (http://www.randomizer.org/). The assessors were blinded to the identity (genetic background or allocation to treatment group) of the animals. Every effort was made to minimize the discomfort and pain of the animals in each phase of the study. Mice were housed in a temperature- and humidity-controlled vivarium (12 h dark/light cycle, free access to food and water, 8 animals per cage). Animals were euthanized with inhaled CO_2_ plus 10–50% O_2_.

### Reagents

HC-030031 [2-(1,3-dimethyl-2,6-dioxo-1,2,3,6-tetrahydro-7H-purin-7-yl)-N-(4-isopropylphenyl) acetamide] was synthesized as previously described^34^. A-967079 [(1E,3E)-1-(4-Fluorophenyl)-2-methyl-1-penten-3-one oxime] was provided by D. Preti (University of Ferrara, Italy). Unless otherwise indicated, all reagents were obtained from Sigma-Aldrich (Milan, Italy).

### IOP injury model

Mice were anesthetized with a mixture of ketamine and xylazine (90 and 3 mg/kg, respectively, intraperitoneal, i.p.) and lightly secured to a platform with wire loops across the upper back and nose. Body temperature was maintained at 37 °C with a homeothermic heating unit to prevent hypothermia during the procedure. Mouse pupil was dilated by adding one drop of a solution of phenylephrine 10% plus tropicamide 0.5% and, for topical anesthesia, one drop of lidocaine hydrochloride (200 mg/ml) was applied to the cornea. Then, the eye was proptosed using curved forceps and the anterior chamber was penetrated with a 30-gauge needle cannulated to a tube infusing sterile isotonic saline (NaCl 0.9%). The IOP was raised to 110 mmHg by elevating the isotonic saline reservoir up to 150 cm above the mouse eye. Ischemia was confirmed by the whitening of the eye. After 60 min of ischemia, the needle was carefully withdrawn, and the reperfusion was confirmed by the return of the normal black color to the iris. The contralateral sham-treated eye was submitted to the same procedure without the IOP increase and was used as control. Drops of sterile phosphate-buffered saline (PBS) were applied to the eye during IOP procedure to maintain cornea lubrification.

Mice were euthanized at day-2 or day-7 after I/R, and the eyes were harvested for immunochemistry/immunofluorescence and molecular analyses. Control groups were statistically similar in every assessment (Supplementary Table 1), supporting a straightforward interpretation of the percentage change scores shown in the Figures.

### Eye drop drug administration

During the days of reperfusion, mice were randomly allocated to the treatment group consisting in eye drop application to the ischemic and nonischemic (control) eye of a solution of A-967079, HC-030031, α-lipoic acid, indomethacin, and 2-acetylphenothiazine (ML171) (5 µl, 10 mM) or their vehicles (all 4% dimethyl sulfoxide, DMSO, 4% tween 80 in NaCl 0.9%). Drugs were first administered 1 h after the ischemia was terminated and then once a day for 2 or 7 consecutive days after reperfusion. Two or 7 days after consecutive administration, both eyes were harvested, and subsequently processed for immunohistochemistry and immunofluorescent analysis.

### Macrophage depletion

To temporarily deplete the monocyte/macrophage population, mice received liposome-encapsulated clodronate (LCL 10 ml/kg of a 5 mg/ml solution, intraperitoneal, ClodronateLiposomes.com, Amsterdam, The Netherlands) or vehicle (liposome-encapsulated phosphate buffer saline) once a day for 3 consecutive days before I/R.

### Immunohistochemistry

Mice were euthanized and the eyes were removed, post-fixed in formalin for 24 h, paraffin embedded and then sectioned (5 µm). Some sections were stained with hematoxylin and eosin (H&E) for histological examination. Other sections were preheated at 50 °C for 20 min, deparaffinized, and then rehydrated in gradients of ethanol (100, 95, 70, and 30%) and water. Antigen retrieval was performed with a citrate buffer solution (pH 6.0) for 20 min at 99 °C. Endogenous peroxidase was blocked by the incubation with a blocking solution (SP-6000, bloxall solution, Vector Laboratories, Burlingame, USA) for 10 min at room temperature. Sections were then incubated with a blocking solution containing 2.5% normal horse serum (PK-7200, Vectastain Elite ABC-HRP Kit, Vector Laboratories, Burlingame, USA), and incubated with the following primary antibodies: RNA-binding protein with multiple splicing (RBPMS, #1832, guinea pig polyclonal, 1:500, PhosphoSolutions, Colorado, USA); glutamic acid decarboxylase (GAD67, #ab26116, mouse monoclonal [K-87], 1:100, Abcam, Cambridge, UK); neuronal nuclei (NeuN, #MAB377, mouse monoclonal [A60], 1:250, Merck, Milan, Italy); F4/80 (#MA516624, rat monoclonal [Cl:A3-1], 1:50, Thermo Fisher Scientific, Rockford, USA) diluted in antibody diluent (Roche Diagnostics, Mannheim, Germany) for 1 h at room temperature. Sections were then incubated with prediluted biotinylated anti-mouse/rabbit IgG secondary antibody or with biotinylated anti-guinea pig secondary antibody (1:200, #BA-7000, Vector Laboratories, Burlingame, USA), 30 min at room temperature, and in avidin-biotin complex solution (#PK-7200, Vectastain Elite ABC-HRP Kit, Vector Laboratories, Burlingame, USA) for 30 min at room temperature. The sections were then transferred to peroxidase substrate (#K3468, ImmPACT DAB, Vector Laboratories, Burlingame, USA) for 4–6 min for the chromogen development reaction and rinsed in distilled water before mounting. Tissues were visualized, and digital images were captured using an optical microscope (Leica DM2500, Leica Microsystems, Milan, Italy).

### Retinal thickness analysis

The retinal thickness (from the internal to the outer limiting membrane) value was obtained as a mean of 5 consecutive fields (40× magnification) of 3 different sections for each eye (for a total of 15 fields analyzed) by using an image processing software (ImageJ 1.32J, NIH, Maryland, USA).

### Cell counting

RBPMS^+^, GAD67^+^, NeuN^+^ cell counting was performed in the ganglion cell layer (GCL) and inner cell layer (INL) and F4/80^+^ cell counting was performed in plexiform layers and in GCL. Cell number was counted in 5 consecutive fields (40× magnification) of 3 different sections for each eye (for a total of 15 fields analyzed).

### Immunofluorescence

Formalin fixed paraffin-embedded sections (5 µm) from mouse or human retinal tissues (#T2234110, BioChain, Newark, CA, USA) and mouse DRGs were deparaffinized, and then rehydrated in gradients of ethanol (100, 95, 70, and 30%) and water. The antigen retrieval was performed with a citrate buffer solution (pH 6.0 or pH 9.0) for 20 min at 99 °C. After blocking 2.5% normal goat serum or 2.5% bovine serum albumin in PBS for 1 h at room temperature, sections were incubated with the following primary antibodies: NeuN (#MAB377, mouse monoclonal [A60], 1:250, Merck, Milan, Italy); TRPA1 (#ARP35205-P050, rabbit polyclonal, 1:300, Aviva Systems Biology, San Diego, USA and #ACC-037, rabbit polyclonal, 1:300, Alomone Labs, Jerusalem, Israel); RBPMS (#1832, guinea pig polyclonal, 1:500, PhosphoSolutions, Colorado, USA); GAD67 (#ab26116, mouse monoclonal [K-87], 1:100, Abcam, Cambridge, UK); Calbindin (#ab82812, mouse monoclonal [CB-955], 1:15, Abcam, Cambridge, UK); glutamine synthetase (GS, #ab64613, mouse monoclonal, 1:300 Abcam, Cambridge, UK); rhodopsin (#ab3267, mouse monoclonal, 1:25, Abcam, Cambridge, UK); active caspase-3 (#ab2302, rabbit polyclonal, 1:25, Abcam, Cambridge, UK); anti-NADPH oxidase 1 (NOX1, #SAB2501686, goat polyclonal, 1:250); 4-HNE (#ab48506, mouse monoclonal [HNEJ-2], 1:40, Abcam, Cambridge, UK) for 1 h at room temperature. Sections were then incubated with fluorescent secondary antibodies: polyclonal Alexa Fluor 488 and polyclonal Alexa Fluor 594 (1:600, Invitrogen, Milan, Italy) 2 h at room temperature, protected from light. Sections were coverslipped using a water-based mounting medium with 4′6′-diamidino-2-phenylindole (DAPI, #ab228549, Abcam, Cambridge, UK). The analysis of negative controls (nonimmune serum) was simultaneously performed to exclude the presence of nonspecific immunofluorescent staining, cross-immunostaining, or fluorescence bleed-through. To confirm the specificity, TRPA1 primary antibodies were preadsorbed (1:1, overnight, 4 °C, or 1 h at room temperature before adding to tissue sections) with their respective antigen peptides (#AAP35205, Aviva Systems Biology, San Diego, USA; #ACC-037 peptide, Alomone Labs, Jerusalem, Israel). Tissues were visualized, and digital images were captured using a Zeiss AxioImager2 microscope in z-stack and Apotome mode (Carl Zeiss Spa, Milan, Italy).

4-HNE and active caspase-3 staining was evaluated as the fluorescence intensity measured by an image processing software (ImageJ 1.32J, NIH, Maryland, USA); data were expressed as mean fluorescence intensity (% of basal). Rhodopsin and GS expression were evaluated measuring the ratio between stained area and total area in 5 consecutive fields (40× magnification) of 3 different sections for each eye. Area quantification and the background removal was performed using the ImageJ software; data were expressed as stained area/total area (% of basal).

Quantification of colocalization was done using Pearson’s correlation (Rcoloc) coefficient obtained by the toolbox analysis JACoP under ImageJ (NIH, Maryland, USA). The number of different cell types expressing TRPA1 was computed as the ratio of TRPA1^+^ cells to the total number of marked cells.

### Real-time PCR

Total RNA was extracted from the retina and DRGs obtained from C57BL/6J mice using the RNeasy Mini kit (Qiagen, Hilden, Germany), according to the manufacturer’s protocol. RNA concentration and purity were assessed spectrophotometrically by measuring the absorbance at 260 and 280 nm. Reverse transcription was performed by the Qiagen QuantiTect Reverse Transcription Kit (Qiagen, Hilden, Germany) following the manufacturer’s protocol. The analysis of TRPA1 mRNA expression in human retina was performed in cDNA from normal human retina tissue (#C1234110-10, BioChain Institute Inc., Newark, USA). The analysis of TRPA1 mRNA expression in human DRG was performed in cDNA obtained from human DRG total RNA (#636150, Takara Bio USA, Inc., Mountain View, USA).

For mRNA relative quantification, real time polymerase chain reaction (PCR) was performed on Rotor Gene^®^ Q (Qiagen, Hilden, Germany). The sets of primers and probes for mouse retina were as follows: Probe1 TRPA1 primer1: 5′-GTACTTCTTGTCGTGTTTCTTGC-3′, primer2: 5′-ACCATCGTGTATCCAAATAGACC-3′, probe: 5′-/56-FAM/AAAACCGTA/ZEN/GCATCCTGCCGTG/3IABkFQ/-3′ (NCBI Ref Seq: NM_177781); Probe2 TRPA1 primer1: 5′-GCTTCCTTTCTGCATATTGCC-3′, primer2: 5′-GATTGGACATCGATTGCTTGG-3′, probe: 5′-/56-FAM/TGTGAGAAC/ZEN/CACTTCCTTGCGCTT/3IABkFQ/-3′ (NCBI Ref Seq: NM_177781); Probe3 TRPA1 primer1: 5′-ATTGTGAATGCAGTTGATGGC-3′, primer2: 5′-AGTGCTGTTGATGTCTGCTC-3′, probe: 5′-/56-FAM/CCCTGCTTC/ZEN/ACAGAGCCTCGTTAT/3IABkFQ/-3′ (NCBI Ref Seq: NM_177781); Rho primer1: 5′-ACCCTCTACACATCACTCCAT-3′, primer2: 5′-CTTGCAGACCACCACGTAG-3′, probe: 5′-/56-FAM/AGAAGCCCT/ZEN/CGAGATTACAGCCTGT/3IABkFQ/-3′ (NCBI Ref Seq: NM_145383); β-actin primer1: 5′-GATTACTGCTCTGGCTCCTAG-3′, primer2: 5′-GACTCATCGTACTCCTGCTTG-3′, probe: 5′-/56-FAM/CTGGCCTCA/ZEN/CTGTCCACCTTCC/3IABkFQ/-3′ (NCBI Ref Seq: NM_007393).

The sets of primers and probes for human retina were as follows: Probe1 TRPA1 primer1: 5′-GAAACCAAAGTGGCAGCTTC-3′, primer2: 5′-GACATTGCTGAGGTCCAGAA-3′, probe: 5′-/56-FAM/TGAAGTTCC/ZEN/ACCTGCATAGCTATCCTCT/3IABkFQ/-3′ (NCBI Ref Seq: NM_007332); Probe2 TRPA1 primer1: 5′-AATCTGCGACCTGAATTTATGC-3′, primer2: 5′-GGACACATTAAAGCCAAGTAGGT-3′, probe: 5′-/56-FAM/ATGTAGAGG/ZEN/AGTACACCCATCGTTGTCT/3IABkFQ/-3′ (NCBI Ref Seq: NM_007332); Probe3 Trpa1 primer1: 5′-TGATGGCTCCTCTCCA-3′, primer2: 5′-GTGTTTCCATTTTCTCCTTCCAA-3′, probe: 5′-/56-FAM/TCACCTCAT/ZEN/TATTCATGCCCTGCACA/3IABkFQ/-3′ (NCBI Ref Seq: NM_007332); Rho primer 1: 5′CGAGGATTCTTGCTTTCTGGA-3′, primer2: 5′-TTCATTCCTCCATTCCTTCCTG-3′, probe: 5′-/56-FAM/ACCTACTGT/ZEN/GTGCCCCATTCTGTG/3IABkFQ/-3′ (NCBI Ref Seq: NM_000539); β-actin primer1: 5′-ACAGAGCCTCGCCTTTG -3′, primer2: 5′-CCTTGCACATGCCGGAG-3′, probe: 5′-/56-FAM/TCATCCATG/ZEN/GTGAGCTGGCGG/3IABkFQ/-3′ (NCBI Ref Seq: NM_001101).

The reference gene for retina was rhodopsin and β-actin for DRG analysis. The QuantiTect Probe PCR Kit (Qiagen, Hilden, Germany) was used for amplification, and processed as follows: samples were heated to 95 °C for 10 min followed by 40 cycles of 95 °C for 5 s, and 60 °C for 20 s. PCR reaction was carried out in triplicate. Relative expression of mRNA was calculated using the 2^−Δ(ΔCT)^ comparative method, with each gene normalized relative to the endogenous reference gene for that sample.

### RNA sequencing

Expression profiles from the Gene Expression Omnibus (GEO) database were used to confirm TRPA1 expression in retina. Due to the limited data on the human retina, we considered all the profile sets available for *mus musculus*. GEO IDs that were considered included: ID:50883559, ID:103901371, ID:50837059, ID:62922359, ID:18760359, ID:62188059, ID:17236959, ID:59616959, ID:18065659, ID:71904759, ID:17132159, ID:126935159, ID:63715659, ID:116907859, ID:126888659, ID:72636259, ID:33032559, and ID:108842871. Of the 18 sets, one (ID:108842871) was removed from the analysis because the expression profile referred to different layers rather than to the whole retina tissue. Ninety-eight samples from the resulting 17 expression profile sets were evaluated. For each sample, genes were rank ordered and sorted according to their expression and then placed into percentile bins. This approach was used to recover the relative activity of TRPA1, compared to other genes, within the considered sample.

### Measurement of A-967079 level in retinal tissue by liquid chromatography–mass spectrometry (LC–MS/MS)

#### Calibration standards and quality control preparation

Two different stock solutions of A-967079 (500 µg/ml) and the internal standard, citalopram (24 µg/ml), were prepared in 100% ethanol and stored at −20 °C. Working solutions (200 and 400 ng/ml) for calibration and controls, respectively, and the internal standard (24 ng/ml), were prepared from the stock solutions by dilution in HPLC grade water. The six points of the calibration curve (0, 8, 20, 40, 80, 100 ng/ml) were prepared by spiking albumin solution (2.5 g/dl) with appropriate amounts of the working solution (200 ng/ml). Quality control samples (16, 48, 80 ng/ml) were prepared separately by adding the working solution (400 ng/ml) in the same way as the calibration standards.

#### Retinal samples preparation

C57BL/6J mice received eye drop instillation with a solution of A-967079 (5 µl, 10 mM, corresponding to a dose of 10.36 µg), and retinas were collected at 5, 10, 15, 30, 60, 180, and 360 min after treatment and homogenized in HPLC grade water. Samples were prepared for the analysis as follows: 60 µl of each sample or each calibration standard (0, 8, 20, 40, 80, 100 ng/ml) or each quality control sample (16, 48, 80 ng/ml) was added with 100 µl 0.3 M sulfate zinc solution diluted (1:5) in methanol and 13 µl of citalopram (24 ng/ml). The resulting mixture was centrifuged (14,000 × *g*, 10 min, room temperature), and surnatant (40 µl) was injected into the LC–MS/MS system. To determine the extraction yield of A-967079, three samples of untreated mice and three samples of albumin solution were spiked with A-967079 (400 ng/ml) before and after the extraction process. The extraction yield was in the 94–100% range. Both samples presented a signal with a similar intensity, confirming no matrix effect.

#### LC–MS/MS

A-967079 levels in retinal samples were measured using high performance liquid chromatography (HPLC LC-20AD XR series system, Shimadzu, Kyoto, Japan) equipped with a binary pump, a degasser, an autosampler and a thermostatic compartment for the chromatographic column. The system was coupled to a QTrap 4000 LC–MS/MS system (ABSciex, Framingham, MA, USA), equipped with a TurboIonSpray source operated in positive ion mode. The ESI configuration was set with ion source temperature 200 °C, curtain gas 30 psi, ion source gas1 55 psi, ion source gas2 60 psi, and ion spray voltage 4000 V. Chromatographic separation was achieved on a Restek Allure PFP propyl (5 μm × 50 mm × 2.1 mm) column set at 40 °C with an operating flow rate of 0.5 ml/min for a total running time of 9 min. Solvent A was 0.2% formic acid and 1 M ammonium formate in water; Solvent B was 0.2% formic acid and 1 M ammonium formate in acetonitrile. The mobile phase was delivered to 10% B in the first 1 min, then in a gradient mode from 10% B to 90% B in 3 min, maintained for 2 min, then from 90% B to 0% B in 0.5 min and re-equilibration for 2.5 min. Analysis by LC–MS/MS was performed in multiple reaction monitoring (MRM) mode with a dwell time of 100 ms, using m/z 208 → 190 and 208 → 176 as qualifier ion and quantifier ion, respectively for A967079 and 325→ 262 for citalopram. Optimal collision cell exit potential was found at 10 V. The resulting declustering potential was +61 V and collision energy were 17, 27, and 20 V for qualifier, quantifier and internal standard, respectively. Data were acquired using Analyst 1.5.1 software and processed using Multiquant 2.1.1 software. Data were expressed as ng/mg of tissue.

### Statistical analysis

Prior to statistical analysis, data for the experimental groups presented in the figures were first transformed to a relative measure of change (%) from the respective control conditions [(EXP–CTL)/CTL*100]. Then, ANOVA was used to compare mean % between different genotypes or treatments. The absolute value of each outcome measure for each of the groups is shown in Table S1; those means were compared using a two-way ANOVA followed by post hoc comparisons using the Bonferroni correction. GraphPad Prism (version 5.00, La Jolla, USA) was used for all analysis and *p* < 0.05 was considered statistically significant. Tabled and charted data represent mean ± standard error of the mean.

## Results

### TRPA1 localizes to mouse and human retinal cells

**Fig. 1:**
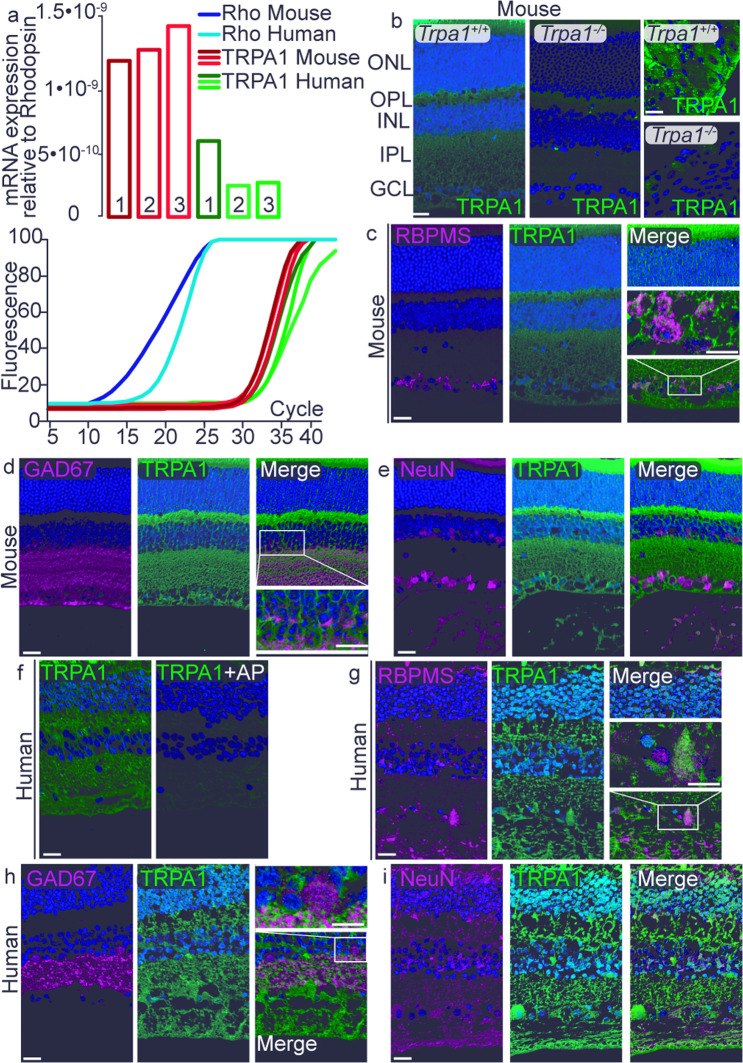
Mouse and human retinal cells express TRPA1. **a** mRNA expression of TRPA1 relative to rhodopsin in mouse and human retina. (1, 2, 3, refer to the 3 different probes for nonoverlapping segments of the cDNA tested). **b** TRPA1 staining in mouse retina and DRG neurons from *Trpa1*^*+/+*^ and *Trpa1*^*−/−*^ mice. **c–e** Double immunofluorescence staining of TRPA1 and RBPMS (**c**), GAD67 (**d**), and NeuN (**e**) in mouse retina. **f** TRPA1 staining in human retina incubated with or without the respective antigen peptide (AP). **g**–**i** Double immunofluorescence staining of TRPA1 and RBPMS (**g**), GAD67 (**h**), and NeuN (**i**) in human retina. Scale bars: 20 µm and inset 10 µm. ONL outer nuclear layer, OPL outer plexiform layer, INL inner nuclear layer, IPL inner plexiform layer, GCL ganglion cell layer. These abbreviations are used for all other figures.

The presence of TRPA1 was assessed by qRT-PCR analysis of retinas taken from C57BL/6J mice and in samples of cDNA taken from normal human retina (Fig. 1a). Three different probes for nonoverlapping cDNA segments of either mouse or human TRPA1 were tested in mouse and human retina, respectively. Results were validated by using cDNA derived from mouse and human dorsal root ganglia (DRG) as positive controls (Supplementary Fig. 1a). In addition, expression profiles from Gene Expression Omnibus (GEO) database were used to confirm TRPA1 presence in the mouse retina, where a low to medium intensity expression level was found (Supplementary Fig. 1b). TRPA1-positive staining was observed throughout the retinal layers and in DRG neurons (control tissue) of wild-type (*Trpa1*^*+/+*^*)* mice, but not in retinas or DRG neurons taken from *Trpa1*^*−/−*^ mice (Fig. 1b; Supplementary Fig. 1c). To identify the specific cell types expressing TRPA1, double staining with antibodies for TRPA1 and cell-specific markers and colocalization by Rcoloc analysis and by estimating the percentage of TRPA1^+^ cells were performed in both mouse and human retina. TRPA1 colocalized with retinal ganglion cells, labeled with the RNA-binding protein with multiple splicing (RBPMS, Fig. 1c; Supplementary Fig. 1f, g), GABAergic amacrine cells, labeled with glutamic acid decarboxylase (GAD67, Fig. 1d; Supplementary Fig. 1f, g), neuronal cells, labeled with the neuronal marker (NeuN, Fig. 1e; Supplementary Fig. 1f, g), Müller glial cells, labeled with glutamine synthetase (GS, Supplementary Fig. 1d, f, g), photoreceptors, labeled with rhodopsin (Supplementary Fig. 1d, f, g) and horizontal cells, labeled with calbindin (Supplementary Fig. 1d, f, g). A pattern of TRPA1 protein expression similar to that observed in mouse was reported in human retina (Fig. 1f–i; Supplementary Fig. 1e–g).

### TRPA1 genetic deletion attenuates retinal damage induced by ischemia/reperfusion

**Fig. 2:**
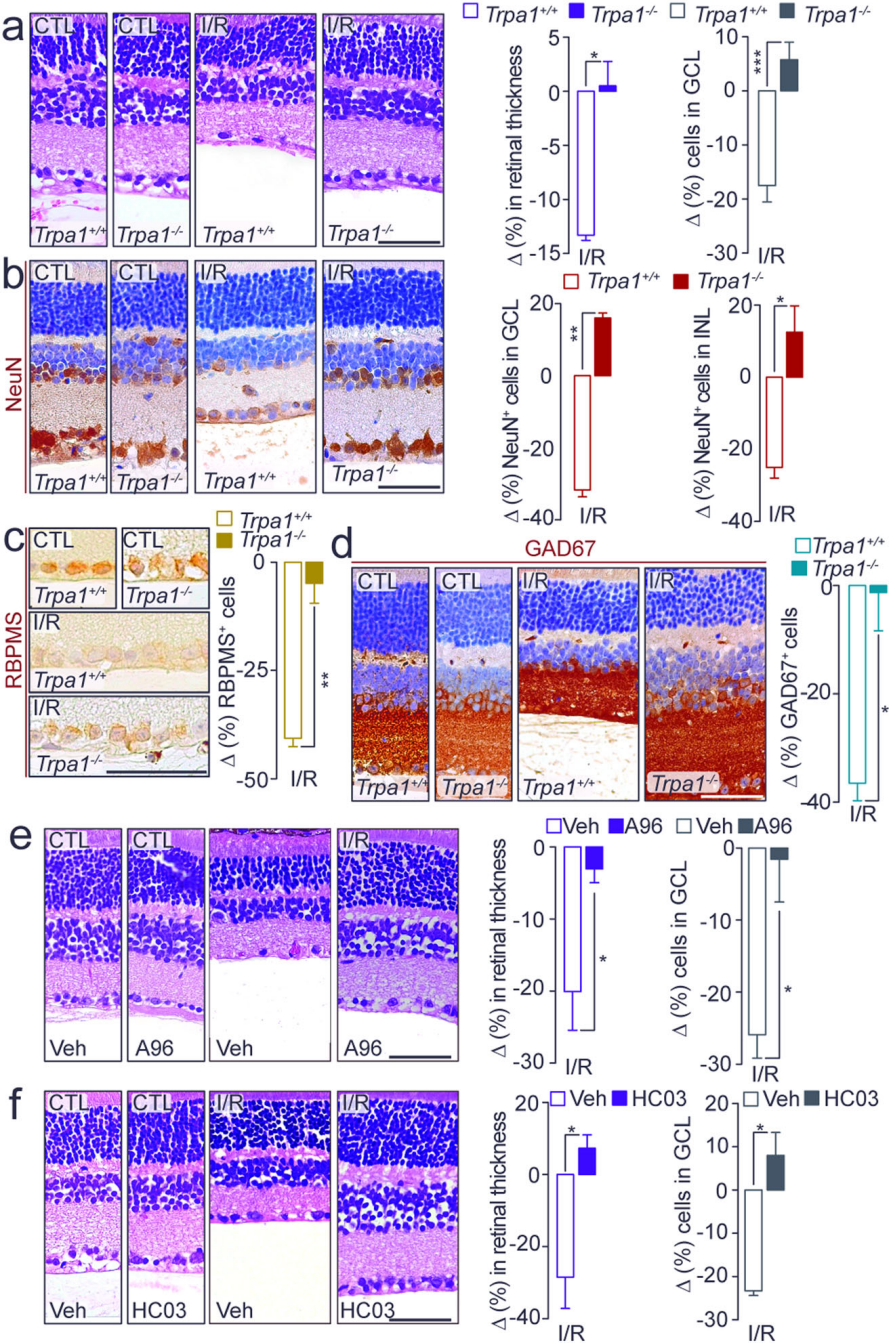
TRPA1 mediates retinal damage. **a** Representative images of hematoxylin and eosin (H&E) staining, retinal thickness and total number of cells in the GCL in retina from *Trpa1*^*+/+*^ and *Trpa1*^*−/−*^ mice after ischemia and reperfusion (I/R). **b** Representative images and total number of NeuN^+^ cells in GCL and INL in retina from *Trpa1*^*+/+*^ and *Trpa1*^*−/−*^ mice after I/R. **c**, **d** Representative images of total number of RBMPS^+^ and GAD67^+^ cells in retina from *Trpa1*^*+/+*^ and *Trpa1*^*−/−*^ mice after I/R. **e**, **f** Representative images of H&E staining, retinal thickness and total number of cells in the GCL in retina from C57BL/6J mice after I/R and treated daily with eye drops (5 µl, 10 mM) A-967079 (A96) (**e**) and HC-030031 (HC03) (**f**) or their vehicle (Veh). All data are from retinas collected at day-7 after I/R. Control (CTL) indicates mice receiving all the procedures except I/R. Scale bars: 50 µm. Data are expressed as the percentage difference (Δ%) from CTL, and displayed as mean ± SEM, *n* = 4 to 5 mice per group. *P < 0.05, **P < 0.01, ***P < 0.001 between indicated groups; two-way ANOVA and Bonferroni post hoc test.

Ischemic insult was provoked by unilateral IOP elevation for 1 h, followed by reperfusion (I/R). Retinas were analyzed at two time points (day-2 and day-7 after I/R), to determine the early and delayed biochemical and cellular changes. In *Trpa1*^*+/+*^ mice, I/R induced a marked decrease in retinal thickness and total number of cells in the ganglion cell layer (GCL) at both day-2 (Supplementary Fig. 2a) and day-7 (Fig. 2a). TRPA1 genetic deletion prevented both effects on both days (day-2, Supplementary Fig. 2a; day-7, Fig. 2a), indicating that TRPA1 is essential in mediating both the early and late effects of I/R injury.

The analysis of tissue damage in specific retinal cells showed a significant reduction in the number of NeuN^+^ cells in GCL and inner nuclear layer (INL) and of RBPMS^+^ cells in GCL and GAD67^+^ cells in INL, both at day-2 (Supplementary Fig. 2b–d) and day-7 (Fig. 2b–d), in *Trpa1*^*+/+*^ mice. The observation that *Trpv1*^*−/−*^ and *Trpv4*^*−/−*^ mice presented a similar reduction in retinal thickness and total cells in GCL compared to their respective wild-type littermates at day-2 after I/R (Supplementary Fig. 3a, b) rules out the role of these channels in retinal damage. Furthermore, *Trpa1*^*−/−*^ mice were protected against the damage of the specific retinal cells (NeuN^+^, RBPMS^+^, and GAD67^+^) both at day-2 (Supplementary Fig. 2b–d) and day-7 (Fig. 2b–d), highlighting the essential role of TRPA1 in the detrimental action of I/R. The analysis of the stained area of GS^+^ and rhodopsin^+^ cells did not show any cell reduction at both day-2 and day-7 after I/R in both *Trpa1*^*+/+*^ and *Trpa1*^*−/−*^ mice (Supplementary Fig. 4).

To investigate the role of TRPA1 expressed in primary sensory neurons, which are particularly abundant in the anterior segment of the eye, mice with selective channel deletion in this cell type (*Adv-Cre;Trpa1*^*fl/fl*^)^32^ were used. At both day-2 and day-7, *Adv-Cre;Trpa1*^*fl/fl*^ mice showed a reduction in retinal thickness and total GCL cell number similar to that found in control mice, indicating that TRPA1 expressed in trigeminal afferents does not contribute to the detrimental effects of I/R (Supplementary Fig. 3c, d).

### A local TRPA1 antagonist attenuates I/R-induced retinal damage

Next, we asked whether TRPA1 antagonism could affect retinal damage in C57BL/6J mice. As shown in *Trpa1*^*+/+*^ mice, in C57BL/6J mice, I/R decreased retinal thickness and total GCL cell number at day-2 (Supplementary Fig. 5a) and day-7 (Fig. 2e). A reduced number of NeuN^+^ cells, both in GCL and INL, and of RBPMS^+^ and GAD67^+^ cells at both time points (Supplementary Fig. 5b–g, i, Supplementary Fig. 5h, j–n) was observed. Daily instillation of eye drops containing two different TRPA1 antagonists, A-967079 or HC-030031, attenuated the reduction in retinal thickness and total GCL cell number (Supplementary Fig. 5a, b; Fig. 2e, f) and in the NeuN^+^ cell number in GCL and INL, and in RBPMS^+^ and GAD67^+^ cells, at day-2 (Supplementary Fig. 5c–g, i) and day-7 (Supplementary Fig. 5h, j–n). LC–MS analysis showed measurable levels of A-967079 in homogenates of mouse retinas that peaked 5 min after eye drop instillation and progressively decreased over time (Supplementary Fig. 5o).

### Oxidative stress and TRPA1-dependent I/R-induced retinal injury

**Fig. 3:**
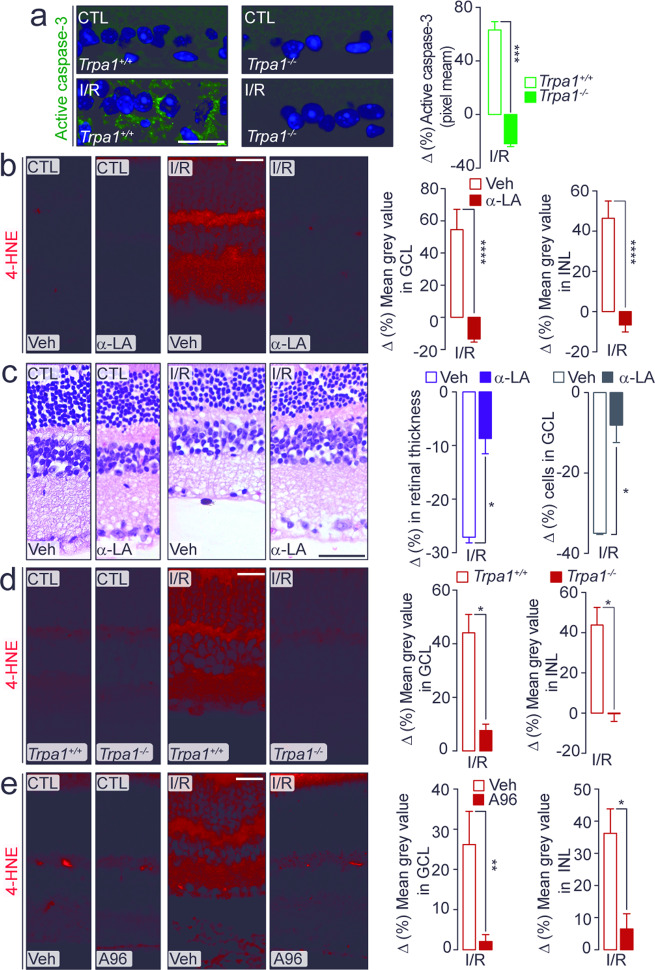
TRPA1 mediates cell death and oxidative stress. **a** Representative images and mean gray value of the active caspase-3 staining in retina from *Trpa1*^*+/+*^ and *Trpa1*^*−/−*^ mice after ischemia and reperfusion (I/R). **b** Representative images and mean gray value of 4-HNE staining in GCL and INL in retina from C57BL/6J mice after I/R and treated daily with eye drops (5 µl, 10 mM) α-lipoic acid (α-LA) or its vehicle (Veh). **c** Representative images of hematoxylin and eosin staining, retinal thickness and total number of cells in the GCL in retina from C57BL/6J mice after I/R and treated daily with eye drops (5 µl, 10 mM) α-LA or its Veh. **d**, **e** Representative images and mean gray value of 4-HNE staining in GCL and INL in retina from (**d**) *Trpa1*^*+/+*^ and *Trpa1*^*−/−*^ after I/R and (**e**) C57BL/6J mice after I/R and treated daily with eye drops (5 µl, 10 mM) A-967079 (A96) or its Veh. All data are from retinas collected at day-7 after I/R. Control (CTL) indicates mice receiving all the procedures except I/R. Scale bars: 20 µm (**a**, **b**, **d**, **e**); 50 µm (**c**). Data are expressed as the percentage difference (Δ%) from CTL, and displayed as mean ± SEM, *n* = 4 to 5 mice per group. *P < 0.05, **P < 0.01, ***P < 0.001, ****P < 0.0001 between indicated groups; two-way ANOVA and Bonferroni post hoc test.

To explore the TRPA1-dependent pathway implicated in retinal damage after I/R, we compared the expression of the active caspase-3, the executor protease of apoptosis activated following ischemic insults^35^, in retinas taken from *Trpa1*^*+/+*^ and *Trpa1*^*−/−*^ mice. The increase in active caspase-3 staining observed both at day-2 (Supplementary Fig. 6a) and day-7 (Fig. 3a), after I/R in the GCL of *Trpa1*^*+/+*^ and C57BL/6J mice, was attenuated in the GCL of *Trpa1*^*−/−*^ mice (Supplementary Fig. 6a; Fig. 3a) and in mice treated daily with A-967079 or HC-030031, both at day-2 (Supplementary Fig. 6b, d) and day-7 (Supplementary Fig. 6c, e) after I/R. Thus, inactivation of the TRPA1 pathway also inactivates caspase-3.

**Fig. 4:**
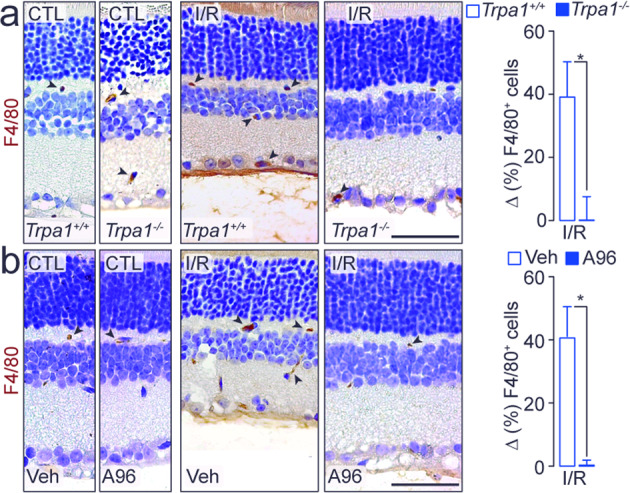
TRPA1 mediates inflammation in damaged retinal tissue. **a**, **b** Representative images and total number of F4/80^+^ cells in retina from (**a**) *Trpa1*^*+/+*^ and *Trpa1*^*−/−*^ mice after ischemia and reperfusion (I/R) and **b** C57BL/6J mice after I/R and treated daily with eye drops (5 µl, 10 mM) A-967079 (A96) or its vehicle (Veh). All data are from retinas collected at day-7 after I/R. Control (CTL) indicates mice receiving all the procedures except I/R. Scale bar: 50 µm. Arrowheads, F4/80^+^ cells. Data are expressed as the percentage difference (Δ%) from CTL, and displayed as mean ± SEM, *n* = 5 mice per group. *P < 0.05 between indicated groups; two-way ANOVA and Bonferroni post hoc test.

Since oxidative stress is critically involved in retinal damage evoked by I/R and increased levels of oxidants have been reported in retinal tissue^8–11^, we evaluated the presence of the oxidative stress end-product, 4-HNE, in the mouse retina. Increased 4-HNE staining was found in both GCL and INL of C57BL/6J mice at day-2 and day-7 after I/R (Supplementary Fig. 6f; Fig. 3b). Daily local instillation (eye drops) of the antioxidant, α-lipoic acid (α-LA), attenuated the increase in 4-HNE staining (Supplementary Figs. 6f and 3b). However, the nonsteroidal anti-inflammatory drug, indomethacin, was ineffective (Supplementary Fig. 6g). α-LA also attenuated the reduction in retinal thickness and in the total cell number in the GCL and RBPMS^+^ cells at day-2 (Supplementary Fig. 7a–c) and day-7 (Fig. 3c) after I/R. Surprisingly, TRPA1 genetic deletion or pharmacological blockade attenuated the increased 4-HNE staining in the retinal GCL and INL (Fig. 3d, e, Supplementary Fig. 7d–g). Tissue injury is usually associated with infiltration of activated macrophages^36^, which contribute to the oxidative burden. The increased number of F4/80^+^ cells observed in the retina of *Trpa1*^*+/+*^ and C57BL/6J mice at day-2 (Supplementary Fig. 8a–c) and day-7 (Fig. 4a, b; Supplementary Fig. 8d) after I/R was reduced in retinas from *Trpa1*^*−/−*^ mice (Fig. 4a; Supplementary Fig. 8a), or in mice receiving daily eye drops of A-967079 or HC-030031 (Fig. 4b and Supplementary Fig. 8b–d). The monocyte/macrophage depleting agent, liposome-encapsulated clodronate, reversed the increase in the number of macrophages in the injured retinas (Supplementary Fig. 8e), but did not affect the reduction in retinal thickness and the increase in 4-HNE staining and caspase-3 activity (Supplementary Fig. 8f–h).

**Fig. 5:**
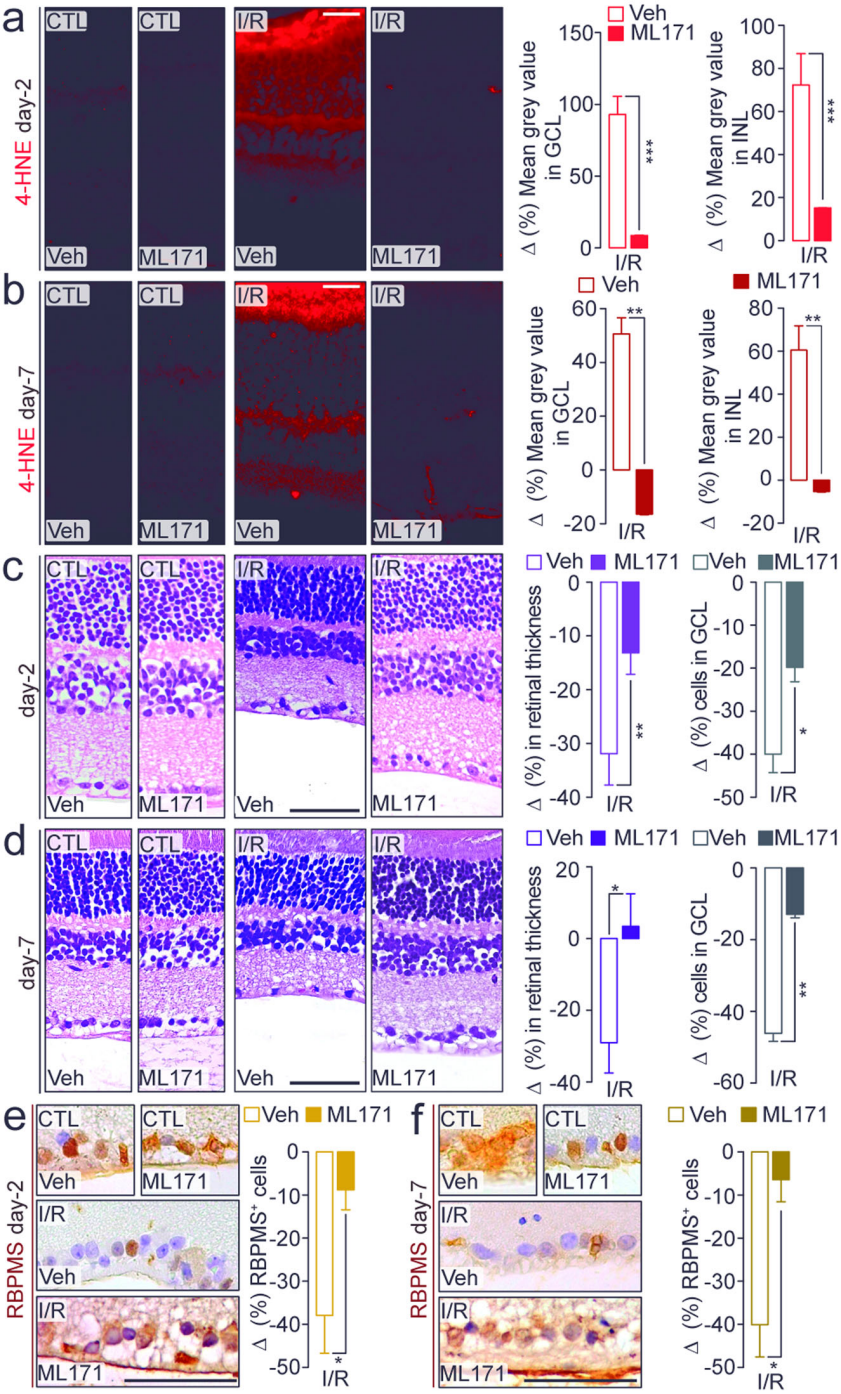
TRPA1/NOX1 pathway is implicated in retinal damage. **a**, **b** Representative images and mean gray value of 4-HNE staining in GCL and INL in retina from C57BL/6J mice after at **a** day-2 and **b** day-7 after ischemia and reperfusion (I/R) and treated daily with eye drops (5 µl, 10 mM) ML171 or its vehicle (Veh). **c**, **d** Representative images of hematoxylin and eosin staining, retinal thickness and total number of cells in the GCL in retina from C57BL/6J mice at **c** day-2 and **d** day-7 after I/R and treated daily with eye drops (5 µl, 10 mM) ML171 or its Veh. Representative images and total number of RBMPS^+^ cells in retina from C57BL/6J mice at **e** day-2 and **f** day-7 after I/R and treated daily with eye drops (5 µl, 10 mM) ML171 or its Veh. Control (CTL) indicates mice receiving all the procedures except I/R. Scale bars: 20 µm (**a**, **b**), 50 µm (**c–f**). Data are expressed as the percentage difference (Δ%) from CTL, and displayed as mean ± SEM, *n* = 4 to 5 mice per group. *P < 0.05, **P < 0.01, ***P < 0.001 between indicated groups; two-way ANOVA and Bonferroni post hoc test.

To explore the pathway by which TRPA1 generates the oxidative stress that sustains the I/R-evoked damage, we localized NOX1 protein in the mouse retina (Supplementary Fig. 9; Supplementary Fig. 1f). NOX1 staining colocalized with TRPA1^+^ cells and was widely found in GS^+^, rhodopsin^+^, calbindin^+^, GAD67^+^, and RBPMS^+^ cells (Supplementary Fig. 9). Furthermore, daily local instillation (eye drops) of the NOX1 inhibitor, ML171, reduced 4-HNE staining in retinal GCL and INL (Fig. 5a, b) and attenuated the reduction in retinal thickness and the number of total cells in GCL and RBPMS^+^ cells, both at day-2 and day-7 after I/R (Fig. 5c–f).

## Discussion

The presence of various TRP channels in the retina and their importance in vision pathophysiology and other retinal functions have been reported^37–40^. Studies on the role of TRPV1 in models of retinal damage have led to conflicting results, probably due to the different types of applied insult. Whereas TRPV1 activation in retinal explants has been reported to promote cell death, and its inhibition to protect retinal ganglion cells from a hydrostatic pressure insult^41,42^, pharmacological inhibition or genetic deletion of this channel has also been reported to exacerbate the IOP-related damage after 5 weeks^43^. TRPV4 expression has also been associated with increased excitability in isolated retinal ganglion cells and retinal explants in response to membrane stretch, resulting in intracellular calcium mobilization and cell death^37^. However, neither *Trpv1*^*−/−*^ nor *Trpv4*^*−/−*^ mice were protected against these ill effects. These data do not support a role for these two channels in mediating the current mouse model of I/R-evoked retinal injury.

We previously observed that TRPA1 is expressed in the avascular chick retina, where its pharmacological inhibition attenuated the production of lactate dehydrogenase evoked by oxygen and glucose deprivation, suggesting a protective role^29^. The present data, using genetic deletion of TRPA1, provide evidence for the first time of the essential role of TRPA1 in the damage caused by I/R in the mouse retina, which, like the human retina, is markedly vascularized. Local TRPA1 antagonists reproduced the knockout phenotype of protection. These findings raise the questions of which TRPA1-expressing cells orchestrate the damaging process and how TRPA1 sustains the I/R-evoked injury. In the anterior eye, TRPA1 is largely present in trigeminal afferents and possibly in other cells^44^. We did not study the role of non-neuronal cells of the anterior eye because we were unable to selectively disrupt the function of TRPA1 in these cells. However, the absence of protection in *Adv-Cre;Trpa1*^*fl/fl*^ mice, which harbor a selective deletion of TRPA1 in primary sensory neurons^32^, excludes a role for these neurons in mediating I/R-evoked damage. Rather, qRT-PCR and immunofluorescence studies showing TRPA1 in a variety of retinal cells, such as Müller glia cells, photoreceptors, horizontal cells, amacrine cells, and ganglion cells, point to these cells as the major contributors of the TRPA1-dependent injury. The observation that elevated levels of A-967079 were measured in the mouse retina after eye drop instillation further supports the hypothesis that the protective action of the TRPA1 antagonist occurs at this anatomical site.

Retinal I/R has been reported to reduce axon density^45^ and cell number^46–49^, thus thinning the optical nerve and decreasing retinal thickness, respectively. In contrast with the damage evoked by transient global ischemia, where retinal alterations are not observed at day-2^50^, the I/R model is characterized by a severe and aggressive reduction in retinal thickness and total cell number in the GCL and NeuN^+^ cells at day-2^3,51^. As reported in a similar model of IOP^46,52^, the robust increase in active caspase-3 in the GCL suggests that an apoptotic process caused the loss of ganglion cells.

Ischemic tissues show increased levels of oxidative stress^53^, which provide a range of actions spanning from cell-to-cell signaling, to cell death and tissue damage^54^. It is known that increased IOP promotes oxidative stress and severe damage in the retina^8^. TRPA1 is markedly sensitive to the redox state of the tissue^19^ and is considered a sensor of oxidative stress^26^. The oxidative stress biomarker, 4-HNE, was increased following I/R in both the GCL and INL of the mouse retina. Protection by daily instillation of eye drops containing the antioxidant, α-LA, or the NOX1 inhibitor, ML171, highlights the role of oxidative stress and NOX1 in the I/R-evoked cell death and retinal damage. Macrophages are recruited at sites of inflammation and tissue injury, including those provoked by ischemia and reperfusion^52,55,56^. In the present model of retinal injury, F4/80^+^ cells were increased in various retinal layers. The ability of the monocyte depleting agent, clodronate, to reverse accumulation of F4/80^+^ cells suggests a hematogenic source of these inflammatory cells.

The finding that both TRPA1 and oxidative stress play an essential role in evoking apoptosis and ensuing cell and tissue loss raises the question on how they contribute to the retinal damaging process. Surprisingly, we observed that the increased 4-HNE levels evoked by I/R were attenuated in *Trpa1*^*−/−*^ mice and in mice treated daily with two different TRPA1 antagonists, A-967079 or HC-030031. Similar unexpected findings were that *Trpa1*^*−/−*^ mice or mice treated with the two TRPA1 antagonists did not show F4/80^+^ accumulation and increase in caspase-3 activity. To explain this data, we advance the most parsimonious hypothesis, which is that I/R activates TRPA1 in one or more retinal cell type(s), where, via a NOX1-dependent pathway, it increases the oxidative burden that sustains the damaging process in the retinal tissue. This pathway shows some similarity with that recently reported in a mouse model of neuropathic pain caused by partial sciatic nerve ligation^28^. TRPA1 in Schwann cells ensheathing the damaged peripheral nerve trunk is essential to sense oxidative stress evoked by infiltrating macrophages and to amplify the oxidative stress signal that sustains neuroinflammation and neuropathic pain^28^. Notably, monocyte depletion by clodronate attenuated F4/80^+^ cell accumulation in the mouse retina, but it did not affect increases in caspase-3 activity and 4-HNE levels and, more importantly, retinal injury. This observation implies that in the I/R model of retinal damage, macrophages are recruited for their conventional function of removing dead cells and debris, and do not have a role in initiating the TRPA1-dependent tissue-damaging pathway, as they do in the sciatic nerve injury model (28).

The remarkable correspondence between the distribution of TRPA1 in mouse and human retina suggests that TRPA1 may play a major role in the damage evoked by ischemic events affecting the human eye. The beneficial effect of the pharmacological treatment with daily eye drops containing a TRPA1 antagonists against I/R-evoked damage in the vascularized mouse retina suggests that such noninvasive therapeutic approaches might be explored in human diseases associated with ischemic retinal damage.

## Supplementary information

Supplemental Figure Legends

Supplementary Table1

Supplemental Figure 1

Supplemental Figure2

Supplemental Figure 3

Supplemental Figure 4

Supplemental Figure 5

Supplemental Figure 6

Supplemental Figure 7

Supplemental Figure 8

Supplemental Figure 9
