# Supplementary material for: TRPA1 mediates damage of the retina induced by ischemia and reperfusion in mice
Source: Cell Death Dis. 2020 Aug 15;11(8):633. doi: 10.1038/s41419-020-02863-6 (PMC7429961; doi:10.1038/s41419-020-02863-6)
Supplement: Supplementary file 1 — Supplemental Figure Legends [file 41419_2020_2863_MOESM1_ESM.docx]

**TRPA1 mediates damage of the retina induced by**

**ischemia and reperfusion in mice**

Daniel Souza Monteiro de Araújo^1,2*^, Francesco De Logu^2*^, Chiara Adembri^2^,

Stanislao Rizzo^3^, Malvin N. Janal^4^, Lorenzo Landini^2^, Alberto Magi^5^, Gianluca Mattei^5^, Nicoletta Cini^6^, Pablo Pandolfo^1^, Pierangelo Geppetti^2^, Romina Nassini^2§^, Karin da Costa Calaza^1^.

^1^Department of Neurobiology and Program of Neurosciences, Institute of Biology, Fluminense Federal University, Niterói, Brazil; ^2^Department of Health Sciences, Section of Clinical Pharmacology and Oncology, University of Florence, Florence, Italy; ^3^Department of Neurosciences, Psychology, Drug Research and Child Health (NeuroFarBa), Division of Ophthalmology, University of Florence, Florence, Italy; ^4^Department of Epidemiology and Health Promotion, New York University College of Dentistry, New York, USA; ^5^Department of Information Engineering, University of Florence, Florence, Italy; ^6^General Laboratory, Careggi University Hospital, Florence, Italy.

^*^D.S.M.A. and F.D.L. contributed equally to this study

^§^**Corresponding author:**

Romina Nassini, PhD

Department of Health Sciences

University of Florence

Viale Pieraccini 6, 50139 Florence, Italy

[romina.nassini@unifi.it](mailto:romina.nassini@unifi.it)

Phone: +39 055 275 8130

Supplemental Fig. 1. (a) mRNA expression of TRPA1 relative to β-actin in mouse and human cDNA from dorsal root ganglion (DRG) (1,2,3, refer to the 3 different probes for non-overlapping segments of the human and mouse TRPA1 cDNA tested) and schematic representation of probe target region of human and mouse TRPA1. (b) Frequency and density plot of the TRPA1 rank distribution in retinal cells. (c) TRPA1 staining in retina and DRG from *Trpa1^+/+^* and *Trpa1^-/-^* mouse and human retina preincubated with or without the antigen peptide (AP). Double immunofluorescence staining of TRPA1 and GS, rhodopsin and calbindin in (d) C57BL/6J mouse and (e) human retina. (f) Pooled data of the colocalization of TRPA1 and NOX1 and the different cell types in mouse and human retina. (g) Pooled data of the TRPA1^+^ cells over the total number of marked cells in mouse and human retina. Scale bars: 20 µm and inset 10 µm.

**Supplemental Fig. 2.** TRPA1 mediates retinal damage. (a) Representative images of hematoxylin and eosin staining, retinal thickness and total number of cells in the GCL in retina from *Trpa1^+/+^* and *Trpa1^-/-^* mice after ischemia and reperfusion (I/R). (b-d) Representative images and total number of (b) NeuN^+^ cells in GCL and INL, and (c) RBMPS^+^ and (d) GAD67^+^ cells in retina from *Trpa1^+/+^* and *Trpa1^-/-^* mice after I/R. All data are from retinas collected at day-2 after I/R. Control (CTL) indicates mice receiving all the procedures except I/R. Scale bars: 50 µm. Data are expressed as the percentage difference (Δ%) from CTL, and displayed as mean ± SEM, n = 4 to 5 mice per group. *P < 0.05, **P < 0.01 between indicated groups; two-way ANOVA and Bonferroni post hoc test.

**Supplemental Fig. 3.** TRPV1, TRPV4, and sensory neuron TRPA1 are not implicated in retinal protection. Representative images of hematoxylin and eosin staining, retinal thickness and total number of cells in the GCL in retina from (a) *Trpv1^+/+^* and *Trpv1^-/-^*, (b) *Trpv4^+/+^* and *Trpv4^-/-^* mice at day-2 after ischemia and reperfusion (I/R) and *Adv-Cre;Trpa1^fl/fl^* and control mice at (c) day-2 and (d) day-7 after I/R. Control (CTL) indicates mice receiving all the procedures except I/R. Scale bars: 50 µm. Data are expressed as the percentage difference (Δ%) from CTL, and displayed as mean ± SEM, n = 4 to 5 mice per group.

**Supplemental Fig. 4.** TRPA1 does not affect photoreceptor damage and the increase in reactive gliosis. (a) Representative images and total area of staining of rhodopsin^+^ cells in retina from *Trpa1^+/+^* and *Trpa1^-/-^* mice after ischemia reperfusion (I/R). (b) Representative images and mean grey value of GS staining in retina from *Trpa1^+/+^* and *Trpa1^-/-^* mice after I/R. All data are from retinas collected at day-2 and day-7 after I/R. Control (CTL) indicates mice receiving all the procedures except I/R. Scale bars: 20 µm. Data are expressed as the percentage difference (Δ%) from CTL, and displayed as mean ± SEM, n = 4 mice per group.

**Supplemental Fig. 5.** TRPA1 pathway is implicated in retinal damage. (a and b) Representative images of hematoxylin and eosin (H&E) staining, retinal thickness and total number of cells in the GCL in retina from C57BL/6J mice, after ischemia and reperfusion (I/R) and treated daily with eye drops (5 µl, 10 mM) A-967079 (A96) and HC-030031 (HC03) or vehicle (Veh). Representative images and total number of (c, d, h, k) NeuN^+^ cells in GCL and INL, (e, g, j, m) RBMPS^+^ cells and (f, i, l, n) GAD67^+^cells in retina from C57BL/6J mice at (a-g and i) day-2 and (h and j-n) day-7 after I/R and treated daily with eye drops (5 µl, 10 mM) HC03 or its Veh. (o) Time course of the retinal concentration of A-967079 after eye drop application (5 µl, 10 mM). Control (CTL) indicates mice receiving all the procedures except I/R. Scale bars: 50 µm. Data are expressed as the percentage difference (Δ%) from CTL, and displayed as mean ± SEM, n = 4 to 5 mice per group. *P < 0.05, **P < 0.01, ***P < 0.001 between indicated groups; two-way ANOVA and Bonferroni post hoc test.

**Supplemental Fig. 6.** TRPA1 pathway is implicated in retinal damage. Representative images and mean grey value of active caspase-3 staining in retina from (a) *Trpa1^+/+^* and *Trpa1^-/-^* mice after ischemia and 2 days of reperfusion (I/R), and from C57BL/6J mice at (b and d) day-2 and (c and e) day-7 after I/R and treated daily with eye drops (5 µl, 10 mM) of A-967079 (A96) or HC-030031 (HC03) or its vehicle (Veh). Representative images and mean grey value of 4-HNE staining in GCL and INL in retina from C57BL/6J mice collected at day-2 after I/R and treated daily with eye drops (5 µl, 10 mM) (d) α-lipoic acid (α-LA) or (e) indomethacin (Indo) or respective Veh. Control (CTL) indicates mice receiving all the procedures except I/R. Scale bars: 20 µm. Data are expressed as the percentage difference (Δ%) from CTL, and displayed as mean ± SEM, n = 4 to 5 mice per group. *P < 0.05, **P < 0.01, ***P < 0.001 between indicated groups; two-way ANOVA and Bonferroni post hoc test.

**Supplemental Fig. 7.** TRPA1 mediates oxidative stress damage. (a) Representative images of hematoxylin and eosin staining, retinal thickness and total number of cells in the GCL in retina from C57BL/6J collected at day-2 after ischemia and reperfusion (I/R) and treated daily with eye drops (5 µl, 10 mM) α-lipoic acid (α-LA) or its vehicle (Veh). (b,c) Representative images and total number of RBPMS^+^ cells in retina from C57BL/6J mice after ischemia and (b) day-2 and (c) day-7 of reperfusion and treated daily with eye drops (5 µl, 10 mM) α-LA or its Veh. (d, e, f, g) Representative images and mean grey value of 4-HNE staining in GCL and INL in retina from *Trpa1^+/+^* and *Trpa1^-/-^* mice and C57BL/6J mice at day-2 after I/R treated daily with eye drops (5 µl, 10 mM) A-967079 (A96) and at day-2 and day-7 after I/R with HC-030031 (HC-03) or Veh. Control (CTL) indicates mice receiving all the procedures except I/R. Data are expressed as the percentage difference (Δ%) from CTL, and displayed as mean ± SEM, n = 4 to 5 mice per group. *P < 0.05, ***P < 0.001 between indicated groups; two-way ANOVA and Bonferroni post hoc test.

**Supplemental Fig. 8.** TRPA1 mediates inflammation in damaged retinal tissue. Representative images and total number of F4/80^+^ cells in retina from (a) *Trpa1^+/+^* and *Trpa1^-/-^* mice, (b and c) C57BL/6J mice at day-2 after ischemia and reperfusion (I/R) and treated daily with eye drops (5 µl, 10 mM) A-967079 (A96) and HC-030031 (HC03) or vehicle (Veh) and (d) C57BL/6J mice at day-7 after I/R and treated daily with eye drops of HC03 or Veh. (e) Representative images and total number of F4/80^+^ cells in retina from C57BL/6J mice treated with liposome-encapsulated clodronate (LCL) or vehicle (PBS). (f) Representative images of hematoxylin and eosin and retinal thickness from C57BL/6J treated with LCL or PBS. (g) Representative images and mean grey value of 4-HNE staining in GCL and INL in retina from C57BL/6J mice treated with LCL or PBS. (h) Representative images and mean grey value of active caspase-3 staining in retina from C57BL/6J mice treated with LCL or PBS. Control (CTL) indicates mice receiving all the procedures except I/R. Scale bar: 50 µm. Arrowheads, F4/80^+^ cells. Data are expressed as the percentage difference (Δ%) from CTL, and displayed as mean ± SEM, n = 4 to 5 mice per group. *P < 0.05, ***P < 0.001 between indicated groups; two-way ANOVA and Bonferroni post hoc test.

**Supplemental Fig. 9.** Double immunofluorescence staining of NOX1 and GS, rhodopsin, calbindin, GAD67, RBMPS and TRPA1 in retinal tissue from C57BL/6J mice. Scale bar: 20 µm and insets 10 µm.
