# Supplementary material for: TRPA1 mediates damage of the retina induced by ischemia and reperfusion in mice
Source: Cell Death Dis. 2020 Aug 15;11(8):633. doi: 10.1038/s41419-020-02863-6 (PMC7429961; doi:10.1038/s41419-020-02863-6)
Supplement: Supplementary file 2 — Supplementary Table1 [file 41419_2020_2863_MOESM2_ESM.docx]

**Supplemental Table 1** Absolute values [mean ± SEM (number of mice per group)] of data reported as % in corresponding figures, supplementary figures and panels.

| **Fig. 2** | | | | |
| --- | --- | --- | --- | --- |
| Fig. 2a | **Retinal thickness (µm)** | | | |
|  | CTL (*Trpa1^+/+^*) | CTL (*Trpa1^-/^*^-^) | I/R (*Trpa1^+/+^*) | I/R (*Trpa1^-/-^*) |
|  | 155.70±7.02(5) | 156.50±6.60(5) | 135.00±0.76(5)* | 157.20±3.55(5)^#^ |
|  | **Total cells (n) in GCL** | | | |
|  | CTL (*Trpa1^+/+^*) | CTL (*Trpa1^-/^*^-^) | I/R (*Trpa1^+/+^*) | I/R (*Trpa1^-/-^*) |
|  | 26.36±0.94(5) | 28.18±0.63(5) | 21.73±0.80(5)** | 27.69±0.42(5)^###^ |
| Fig. 2b | **NeuN^+^ cells (n) in GCL** | | | |
|  | CTL (*Trpa1^+/+^*) | CTL (*Trpa1^-/^*^-^) | I/R (*Trpa1^+/+^*) | I/R (*Trpa1^-/-^*) |
|  | 30.03±0.49(5) | 24.08±1.50(5) | 20.50±0.57(5)*** | 27.93±0.33(5)^##^ |
|  | **NeuN^+^ cells (n) in INL** | | | |
|  | CTL (*Trpa1^+/+^*) | CTL (*Trpa1^-/^*^-^) | I/R (*Trpa1^+/+^*) | I/R (*Trpa1^-/-^*) |
|  | 68.65±6.03(5) | 57.16±5.43(5) | 49.43±1.35(5)* | 67.28±3.67(5)^#^ |
| Fig. 2c | **RBPMS^+^ cells (n)** | | | |
|  | CTL (*Trpa1^+/+^*) | CTL (*Trpa1^-/^*^-^) | I/R (*Trpa1^+/+^*) | I/R (*Trpa1^-/-^*) |
|  | 29.29±0.80(4) | 28.67±1.27(4) | 17.38±0.56(4)* | 27.25±1.32(4)^##^ |
| Fig. 2d | **GAD67^+^ cells (n)** | | | |
|  | CTL (*Trpa1^+/+^*) | CTL (*Trpa1^-/^*^-^) | I/R (*Trpa1^+/+^*) | I/R (*Trpa1^-/-^*) |
|  | 95.14±5.04(4) | 95.50±9.27(4) | 60.38±3.06(4)* | 94.25±6.75(4)^#^ |
| Fig. 2e | **Retinal thickness (µm)** | | | |
|  | CTL Veh | CTL A96 | I/R Veh | I/R A96 |
|  | 135.16±3.32(4) | 138.40±4.17(4) | 107.38±3.06(4)* | 133.50±1.14(4)^#^ |
|  | **Total cells (n) in GCL** | | | |
|  | CTL Veh | CTL A96 | I/R Veh | I/R A96 |
|  | 29.55±1.33(4) | 29.07±0.99(4) | 21.75±0.88(4)** | 28.48±1.65(4)^#^ |
| Fig. 2f | **Retinal thickness (µm)** | | | |
|  | CTL Veh | CTL HC03 | I/R Veh | I/R HC03 |
|  | 154.50±2.12(4) | 148.10±14.74(4) | 107.10±12.04(4)* | 158.80±5.59(4)^#^ |
|  | **Total cells (n) in GCL** | | | |
|  | CTL Veh | CTL HC03 | I/R Veh | I/R HC03 |
|  | 26.97±1.62(4) | 25.02±0.48(4) | 20.67±0.29(4)* | 27.02±1.33(4)^#^ |
| *P<0.05, **P<0.01, ***P<0.001 *vs.* CTL (*Trpa1^+/+^*) or CTL Veh  ^#^P<0.05, ^##^P<0.01, ^###^P<0.001 *vs.* I/R (*Trpa1^+/+^*) or I/R Veh | | | | |
| **Fig. 3** | | | | |
| Fig. 3a | **Active caspase-3 (mean grey value)** | | | |
|  | CTL (*Trpa1^+/+^*) | CTL (*Trpa1^-/^*^-^) | I/R (*Trpa1^+/+^*) | I/R (*Trpa1^-/-^*) |
|  | 22.88±1.18(5) | 23.75±0.70(5) | 37.33±1.42(5)*** | 18.58±0.49(5)^###^ |
| Fig. 3b | **4-HNE (mean grey value) in GCL** | | | |
|  | CTL Veh | CTL α-LA | I/R Veh | I/R α-LA |
|  | 5.23±0.20(4) | 4.48±0.24(4) | 8.08±0.66(4)*** | 3.88±0.09(4)^####^ |
|  | **4-HNE (mean grey value) in INL** | | | |
|  | CTL Veh | CTL α-LA | I/R Veh | I/R α-LA |
|  | 5.51±0.29(4) | 4.05±0.14(4) | 8.07±0.47(4)*** | 3.78±0.14(4)^###^ |
| Fig. 3c | **Retinal thickness (µm)** | | | |
|  | CTL Veh | CTL α-LA | I/R Veh | I/R α-LA |
|  | 82.19±26(4) | 86.71±7.34(4) | 59.89±0.85(4)* | 79.19±2.49(4)^#^ |
|  | **Total cells (n) in GCL** | | | |
|  | CTL Veh | CTL α-LA | I/R Veh | I/R α-LA |
|  | 25.61±0.58(4) | 22.62±1.13(4) | 16.64±0.07(4)*** | 20.78±0.98(4)^#^ |
| Fig. 3d | **4-HNE (mean grey value) in GCL** | | | |
|  | CTL (*Trpa1^+/+^*) | CTL (*Trpa1^-/^*^-^) | I/R (*Trpa1^+/+^*) | I/R (*Trpa1^-/-^*) |
|  | 5.19±0.27(4) | 5.30±0.35(4) | 7.48±0.36(4)* | 5.71±0.13(4)^#^ |
|  | **4-HNE (mean grey value) in INL** | | | |
|  | CTL (*Trpa1^+/+^*) | CTL (*Trpa1^-/^*^-^) | I/R (*Trpa1^+/+^*) | I/R (*Trpa1^-/-^*) |
|  | 5.39±0.38(4) | 5.95±0.56(4) | 7.74±0.47(4)* | 5.88±0.17(4)^#^ |
| Fig. 3e | **4-HNE (mean grey value) in GCL** | | | |
|  | CTL Veh | CTL A96 | I/R Veh | I/R A96 |
|  | 22.66±1.05(4) | 22.36±0.13(4) | 28.75±1.79(4)** | 22.98±0.30(4)^##^ |
|  | **4-HNE (mean grey value) in INL** | | | |
|  | CTL Veh | CTL A96 | I/R Veh | I/R A96 |
|  | 18.43±1.43(4) | 19.36±0.40(4) | 25.27±1.32(4)** | 20.78±0.83(4)^#^ |
| *P<0.05, **P<0.01, ***P<0.001 *vs.* CTL (*Trpa1^+/+^*) or CTL Veh  ^#^P<0.05, ^##^P<0.01, ^###^P<0.001, ^####^P<0.0001 *vs.* I/R (*Trpa1^+/+^*) or I/R Veh | | | | |
| **Fig. 4** | | | | |
| Fig. 4a | **F4/80^+^ cells (n)** | | | |
|  | CTL (*Trpa1^+/+^*) | CTL (*Trpa1^-/^*^-^) | I/R (*Trpa1^+/+^*) | I/R (*Trpa1^-/-^*) |
|  | 12.65±1.08(5) | 12.18±1.34(5) | 17.60±1.42(5)* | 12.19±0.91(5)^#^ |
| Fig. 4b | **F4/80^+^ cells (n)** | | | |
|  | CTL Veh | CTL A96 | I/R Veh | I/R A96 |
|  | 12.67±1.71(4) | 13.02±1.22(4) | 17.94±1.19(4)* | 13.13±0.23(4)^#^ |
| *P<0.0, *vs*. CTL (*Trpa1^+/+^*) or CTL Veh  ^#^P<0.05 *vs.* I/R (*Trpa1^+/+^*) or I/R Veh | | | | |
| **Fig. 5** | | | | |
| Fig. 5a | **4-HNE (mean grey value) in GCL day-2** | | | |
|  | CTL Veh | CTL ML171 | I/R Veh | I/R ML171 |
|  | 8.46±0.74(4) | 6.96±0.89(4) | 16.34±1.07(4)*** | 7.56±0.02(4)^###^ |
|  | **4-HNE (mean grey value) in INL day-2** | | | |
|  | CTL Veh | CTL ML171 | I/R Veh | I/R ML171 |
|  | 7.47±0.36(4) | 6.40±0.47(4) | 12.87±1.09(4)*** | 7.37±0.01(4)^###^ |
| Fig. 5b | **4-HNE (mean grey value) in GCL day-7** | | | |
|  | CTL Veh | CTL ML171 | I/R Veh | I/R ML171 |
|  | 7.98±1.09(4) | 9.26±0.26(4) | 12.02±0.48(4)** | 7.74±0.03(4)^##^ |
|  | **4-HNE (mean grey value) in INL day-7** | | | |
|  | CTL Veh | CTL ML171 | I/R Veh | I/R ML171 |
|  | 6.80±0.64(4) | 7.46±0.34(4) | 10.92±0.76(4)** | 7.07±0.02(4)^##^ |
| Fig. 5c | **Retinal thickness (µm) day-2** | | | |
|  | CTL Veh | CTL ML171 | I/R Veh | I/R ML171 |
|  | 141.80±6.47(4) | 163.60±4.91(4) | 96.58±8.28(4)** | 142.10±6.55(4)^##^ |
|  | **Total cells (n) in GCL day-2** | | | |
|  | CTL Veh | CTL ML171 | I/R Veh | I/R ML171 |
|  | 25.22±1.04(4) | 24.60±0.99(4) | 15.14±1.10(4)*** | 19.73±0.82(4)^#^ |
| Fig. 5d | **Retinal thickness (µm) day-7** | | | |
|  | CTL Veh | CTL ML171 | I/R Veh | I/R ML171 |
|  | 154.50±2.12(4) | 135.90±11.64(4) | 106.2±11.53(4)* | 146.70±8.96(4)^#^ |
|  | **Total cells (n) in GCL day-7** | | | |
|  | CTL Veh | CTL ML171 | I/R Veh | I/R ML171 |
|  | 25.24±1.65(4) | 25.52±0.77(4) | 13.59±0.57(4)**** | 22.20±0.23(4)^##^ |
| Fig. 5e | **RBPMS^+^ cells (n) day-2** | | | |
|  | CTL Veh | CTL ML171 | I/R Veh | I/R ML171 |
|  | 24.33±2.17(4) | 22.43±0.82(4) | 14.58±1.82(4)*** | 21.00±1.15(4)^#^ |
| Fig. 5f | **RBPMS^+^ cells (n) day-7** | | | |
|  | CTL Veh | CTL ML171 | I/R Veh | I/R ML171 |
|  | 25.27±0.94(4) | 22.98±0.94(4) | 15.69±0.84(4)** | 20.97±1.06(4)^#^ |
| *P<0.05, **P<0.01, ***P<0.001, ****P<0.0001 *vs.* CTL (*Trpa1^+/+^*) or CTL Veh  ^#^P<0.05, ^##^P<0.01, ^###^P<0.001 *vs.* I/R (*Trpa1^+/+^*) or I/R Veh | | | | |
| **Supplemental Fig 2** | | | | |
| 2a | **Retinal thickness (µm)** | | | |
|  | CTL (*Trpa1^+/+^*) | CTL (*Trpa1^-/^*^-^) | I/R (*Trpa1^+/+^*) | I/R (*Trpa1^-/-^*) |
|  | 128.00±6.39(5) | 131.90±7.74(5) | 100.60±5.47(5)* | 126.30±7.91(5)^#^ |
|  | **Total cells (n) in GCL** | | | |
|  | CTL (*Trpa1^+/+^*) | CTL (*Trpa1^-/^*^-^) | I/R (*Trpa1^+/+^*) | I/R (*Trpa1^-/-^*) |
|  | 26.63±0.82(5) | 25.12±1.81(5) | 20.28±1.66(5)* | 27.38±1.59(5)^#^ |
| 2b | **NeuN^+^ cells (n) in GCL** | | | |
|  | CTL (*Trpa1^+/+^*) | CTL (*Trpa1^-/^*^-^) | I/R (*Trpa1^+/+^*) | I/R (*Trpa1^-/-^*) |
|  | 27.37±1.46(5) | 28.34±2.14(5) | 19.88±0.22(5)* | 28.88±2.11(5)^#^ |
|  | **NeuN^+^ cells (n) in INL** | | | |
|  | CTL (*Trpa1^+/+^*) | CTL (*Trpa1^-/^*^-^) | I/R (*Trpa1^+/+^*) | I/R (*Trpa1^-/-^*) |
|  | 68.45±3.77(5) | 61.59±7.20(5) | 45.53±1.60(5)* | 69.98±1.66(5)^#^ |
| 2c | **RBPMS^+^ cells (n)** | | | |
|  | CTL (*Trpa1^+/+^*) | CTL (*Trpa1^-/^*^-^) | I/R (*Trpa1^+/+^*) | I/R (*Trpa1^-/-^*) |
|  | 32.81±4.35(4) | 37.52±3.49(4) | 20.10±0.55(4)* | 34.31±3.45(4)^##^ |
| 2d | **GAD67^+^ cells (n)** | | | |
|  | CTL (*Trpa1^+/+^*) | CTL (*Trpa1^-/^*^-^) | I/R (*Trpa1^+/+^*) | I/R (*Trpa1^-/-^*) |
|  | 83.71±3.42(4) | 79.37±2.81(4) | 55.10±4.37(4)* | 75.61±5.87(4)^#^ |
| *P<0.05 *vs.* CTL (*Trpa1^+/+^*)  ^#^P<0.05, ^##^P<0.01 *vs.* I/R (*Trpa1^+/+^*) | | | | |
| **Supplemental Fig 3** | | | | |
| 3a | **Retinal thickness (µm)** | | | |
|  | CTL (*Trpv1^+/+^*) | CTL (*Trpv1^-/^*^-^) | I/R (*Trpv1^+/+^*) | I/R (*Trpv1^-/-^*) |
|  | 81.46±1.64(4) | 84.63±4.52(4) | 64.75±3.25(4)* | 64.56±3.41(4) |
|  | **Total cells (n) in GCL** | | | |
|  | CTL (*Trpv1^+/+^*) | CTL (*Trpv1^-/^*^-^) | I/R (*Trpv1^+/+^*) | I/R (*Trpv1^-/-^*) |
|  | 25.91±0.78(4) | 27.98±0.56(4) | 19.30±1.18(4)** | 18.25±1.32(4) |
| 3b | **Retinal thickness (µm)** | | | |
|  | CTL (*Trpv4^+/+^*) | CTL (*Trpv4^-/^*^-^) | I/R (*Trpv4^+/+^*) | I/R (*Trpv4^-/-^*) |
|  | 81.00±2.53(4) | 78.45±2.37(4) | 66.82±1.42(4)** | 61.40±1.98(4) |
|  | **Total cells (n) in GCL** | | | |
|  | CTL (*Trpv4^+/+^*) | CTL (*Trpv4^-/^*^-^) | I/R (*Trpv4^+/+^*) | I/R (*Trpv4^-/-^*) |
|  | 23.74±1.06(4) | 26.08±0.98(4) | 17.42±0.41(4)** | 17.69±1.10(4) |
| 3c | **Retinal thickness (µm) day-2** | | | |
|  | CTL | CTL (*Adv-Cre*) | I/R | I/R (*Adv-Cre*) |
|  | 75.83±3.63(4) | 67.29±2.79(4) | 61.03±0.82(4)* | 52.96±1.98(4) |
|  | **Total cells (n) in GCL day-2** | | | |
|  | CTL | CTL (*Adv-Cre*) | I/R | I/R (*Adv-Cre*) |
|  | 30.95±1.96(4) | 27.45±1.13(4) | 22.40±1.39(4)** | 20.89±1.36(4) |
| 3d | **Retinal thickness (µm) day-7** | | | |
|  | CTL | CTL (*Adv-Cre*) | I/R | I/R (*Adv-Cre*) |
|  | 71.10±1.69(4) | 70.10±3.34(4) | 60.76±0.63(4)* | 54.51±2.11(4) |
|  | **Total cells (n) in GCL day-7** | | | |
|  | CTL | CTL (*Adv-Cre*) | I/R | I/R (*Adv-Cre*) |
|  | 28.27±1.50(4) | 30.15±1.64(4) | 22.39±0.41(4)* | 23.00±1.07(4) |
| *P<0.05, **P<0.01 *vs.* CTL (*Trpv1^+/+^*), CTL (*Trpv4^+/+^*) or CTL (*Adv-Cre*) | | | | |
| **Supplemental Fig 4** | | | | |
| 4a | **Rhodopsin^+^ area (stained area/total area) day-2** | | | |
|  | CTL (*Trpa1^+/+^*) | CTL (*Trpa1^-/^*^-^) | I/R (*Trpa1^+/+^*) | I/R (*Trpa1^-/-^*) |
|  | 0.34±0.01(4) | 0.29±0.05(4) | 0.30±0.02(4) | 0.28±0.01(4) |
|  | **Rhodopsin^+^ area (stained area/total area) day-7** | | | |
|  | CTL (*Trpa1^+/+^*) | CTL (*Trpa1^-/^*^-^) | I/R (*Trpa1^+/+^*) | I/R (*Trpa1^-/-^*) |
|  | 0.29±0.02(4) | 0.31±0.04(4) | 0.27±0.01(4) | 0.28±0.01(4) |
| 4b | **GS^+^ area (stained area/total area) day-2** | | | |
|  | CTL (*Trpa1^+/+^*) | CTL (*Trpa1^-/^*^-^) | I/R (*Trpa1^+/+^*) | I/R (*Trpa1^-/-^*) |
|  | 0.16±0.03(4) | 0.10±0.01(4) | 0.18±0.11(4) | 0.11±0.02(4) |
|  | **GS^+^ area (stained area/total area) day-7** | | | |
|  | CTL (*Trpa1^+/+^*) | CTL (*Trpa1^-/^*^-^) | I/R (*Trpa1^+/+^*) | I/R (*Trpa1^-/-^*) |
|  | 0.14±0.03(4) | 0.08±0.01(4) | 0.14±0.03(4) | 0.14±0.02(4) |
| **Supplemental Fig 5** | | | | |
| 5a | **Retinal thickness (µm)** | | | |
|  | CTL Veh | CTL A96 | I/R Veh | I/R A96 |
|  | 165.16±3.06(4) | 157.36±2.61(4) | 114.10±5.55(4)** | 150.40±4.15(4)^#^ |
|  | **Total cells (n) in GCL** | | | |
|  | CTL Veh | CTL A96 | I/R Veh | I/R A96 |
|  | 27.89±1.52(4) | 27.79±0.38(4) | 18.15±0.63(4)*** | 26.60±0.47(4)^###^ |
| 5b | **Retinal thickness (µm)** | | | |
|  | CTL Veh | CTL HC03 | I/R Veh | I/R HC03 |
|  | 141.80±6.47(5) | 134.40±9.89(5) | 96.58±8.28(5)* | 125.40±3.20(5)^#^ |
|  | **Total cells (n) in GCL** | | | |
|  | CTL Veh | CTL HC03 | I/R Veh | I/R HC03 |
|  | 26.98±1.91(5) | 25.83±1.00(5) | 16.76±2.21(5)* | 27.80±1.76(5)^#^ |
| 5c | **NeuN^+^ cells (n) in GCL day-2** | | | |
|  | CTL Veh | CTL A96 | I/R Veh | I/R A96 |
|  | 34.75±2.50(4) | 35.88±2.64(4) | 24.94±1.03(4)* | 38.44±2.84(4)^##^ |
|  | **NeuN^+^ cells (n) in INL day-2** | | | |
|  | CTL Veh | CTL A96 | I/R Veh | I/R A96 |
|  | 72.06±9.03(4) | 72.75±2.48(4) | 46.44±0.77(4)* | 73.38±5.08(4)^#^ |
| 5d | **NeuN^+^ cells (n) in GCL day-2** | | | |
|  | CTL Veh | CTL HC03 | I/R Veh | I/R HC03 |
|  | 26.40±1.77(5) | 25.25±1.23(5) | 18.21±1.20(5)* | 26.35±1.35(5)^#^ |
|  | **NeuN^+^ cells (n) in INL day-2** | | | |
|  | CTL Veh | CTL HC03 | I/R Veh | I/R HC03 |
|  | 57.13±4.80(5) | 54.28±3.06(5) | 41.47±3.28(5)* | 59.01±1.51(5)^#^ |
| 5e | **RBPMS^+^ cells (n) day-2** | | | |
|  | CTL Veh | CTL A96 | I/R Veh | I/R A96 |
|  | 27.92±1.34(4) | 30.43±1.83(4) | 19.80±2.31(4)* | 31.40±1.79(4)^##^ |
| 5f | **GAD67^+^ cells (n) day-2** | | | |
|  | CTL Veh | CTL A96 | I/R Veh | I/R A96 |
|  | 98.90±6.11(4) | 101.20±0.99(4) | 69.90±2.66(4)*** | 95.38±1.44(4)^###^ |
| 5g | **RBPMS^+^ cells (n) day-2** | | | |
|  | CTL Veh | CTL HC03 | I/R Veh | I/R HC03 |
|  | 31.25±2.30(4) | 35.13±1.23(4) | 20.70±0.75(4)* | 32.48±2.21(4)^##^ |
| 5h | **NeuN^+^ cells (n) in GCL day-7** | | | |
|  | CTL Veh | CTL A96 | I/R Veh | I/R A96 |
|  | 35.25±1.67(4) | 32.50±0.42(4) | 27.56±1.06(4)** | 37.25±1.17(4)^###^ |
|  | **NeuN^+^ cells (n) in INL day-7** | | | |
|  | CTL Veh | CTL A96 | I/R Veh | I/R A96 |
|  | 66.50±3.92(4) | 65.75±3.65(4) | 50.00±1.49(4)* | 69.25±4.96(4)^#^ |
| 5i | **GAD67^+^ cells (n) day-2** | | | |
|  | CTL Veh | CTL HC03 | I/R Veh | I/R HC03 |
|  | 78.47±2.20(4) | 73.75±2.26(4) | 56.76±3.83(4)* | 76.34±4.81(4)^#^ |
| 5j | **RBPMS^+^ cells (n) day-7** | | | |
|  | CTL Veh | CTL A96 | I/R Veh | I/R A96 |
|  | 30.03±1.81(4) | 30.60±1.01(4) | 21.50±1.12(4)* | 31.48±2.12(4)^##^ |
| 5k | **NeuN^+^ cells (n) in GCL day-7** | | | |
|  | CTL Veh | CTL HC03 | I/R Veh | I/R HC03 |
|  | 28.44±1.79(5) | 22.68±0.84(5) | 18.00±1.15(5)* | 23.01±0.76(5)^#^ |
|  | **NeuN^+^ cells (n) in INL day-7** | | | |
|  | CTL Veh | CTL HC03 | I/R Veh | I/R HC03 |
|  | 40.58±1.42(5) | 38.11±3.50(5) | 24.53±2.16(5)* | 38.58±3.42(5)^#^ |
| 5l | **GAD67^+^ cells (n) day-7** | | | |
|  | CTL Veh | CTL A96 | I/R Veh | I/R A96 |
|  | 98.48±4.18(4) | 110.00±2.77(4) | 74.40±2.70(4)** | 103.00±3.86(4)^###^ |
| 5m | **RBPMS^+^ cells (n) day-7** | | | |
|  | CTL Veh | CTL HC03 | I/R Veh | I/R HC03 |
|  | 27.52±2.36(4) | 30.20±1.05(4) | 20.38±1.16(4)* | 28.49±1.46(4)^#^ |
| 5n | **GAD67^+^ cells (n) day-7** | | | |
|  | CTL Veh | CTL HC03 | I/R Veh | I/R HC03 |
|  | 76.82±5.06(4) | 71.50±0.95(4) | 50.96±3.77(4)** | 67.85±4.55(4)^##^ |
| *P<0.05, **P<0.01, ***P<0.001 *vs.* CTL Veh  ^#^P<0.05, ^##^P<0.01, ^###^P<0.001 *vs.* I/R Veh | | | | |
| **Supplemental Fig 6** | | | | |
| 6a | **Active caspase-3 (mean grey value) day-2** | | | |
|  | CTL (*Trpa1^+/+^*) | CTL (*Trpa1^-/^*^-^) | I/R (*Trpa1^+/+^*) | I/R (*Trpa1^-/-^*) |
|  | 23.07±0.46(5) | 18.22±1.24(5) | 33.36±1.49(5)* | 15.41±0.35(5)^##^ |
| 6b | **Active caspase-3 (mean grey value) day-2** | | | |
|  | CTL Veh | CTL A96 | I/R Veh | I/R A96 |
|  | 19.55±1.12(4) | 18.28±0.61(4) | 28.59±2.05(4)** | 18.04±0.50(4)^###^ |
| 6c | **Active caspase-3 (mean grey value) day-7** | | | |
|  | CTL Veh | CTL A96 | I/R Veh | I/R A96 |
|  | 21.96±0.99(4) | 19.99±1.00(4) | 28.15±0.48(4)* | 19.21±2.41(4)^##^ |
| 6d | **Active caspase-3 (mean grey value) day-2** | | | |
|  | CTL Veh | CTL HC03 | I/R Veh | I/R HC03 |
|  | 17.35±0.90(5) | 15.19±1.03(5) | 25.39±2.37(5)* | 19.00±1.07(5)^#^ |
| 6e | **Active caspase-3 (mean grey value) day-7** | | | |
|  | CTL Veh | CTL HC03 | I/R Veh | I/R HC03 |
|  | 13.76±0.42(5) | 12.83±0.85(5) | 22.81±0.93(5)*** | 13.99±1.52(5)^###^ |
| 6f | **4-HNE (mean grey value) in GCL** | | | |
|  | CTL Veh | CTL α-LA | I/R Veh | I/R α-LA |
|  | 10.81±0.49(4) | 13.00±1.00(4) | 15.97±1.08(4)** | 12.11±0.67(4)^#^ |
|  | **4-HNE (mean grey value) in INL** | | | |
|  | CTL Veh | CTL α-LA | I/R Veh | I/R α-LA |
|  | 11.32±0.45(4) | 13.19±1.08(4) | 16.79±0.89(4)** | 13.45±0.30(4)^#^ |
| 6g | **4-HNE (mean grey value) in GCL** | | | |
|  | CTL Veh | CTL Indo | I/R Veh | I/R Indo |
|  | 13.11±1.53(4) | 11.32±0.81(4) | 18.84±1.25(4)* | 19.89±1.59(4) |
|  | **4-HNE (mean grey value) in INL** | | | |
|  | CTL Veh | CTL Indo | I/R Veh | I/R Indo |
|  | 11.92±0.59(4) | 10.76±0.48(4) | 20.05±1.33(4)* | 17.17±2.04(4) |
| *P<0.05, **P<0.01, ***P<0.001 *vs.* CTL (*Trpa1^+/+^*) or CTL Veh  ^#^P<0.05, ^##^P<0.01, ^###^P<0.001 *vs.* I/R (*Trpa1^+/+^*) or I/R Veh | | | | |
| **Supplemental Fig 7** | | | | |
| 7a | **Retinal thickness (µm) day-2** | | | |
|  | CTL Veh | CTL α-LA | I/R Veh | I/R α-LA |
|  | 79.77±4.06(4) | 75.37±2.49(4) | 64.09±2.02(4)* | 76.34±2.37(4)^#^ |
|  | **Total cells (n) in GCL day-2** | | | |
|  | CTL Veh | CTL α-LA | I/R Veh | I/R α-LA |
|  | 27.57±2.49(4) | 25.29±1.47(4) | 17.58±2.02(4)* | 25.31±0.44(4)^#^ |
| 7b | **RBPMS^+^ cells (n) day-2** | | | |
|  | CTL Veh | CTL α-LA | I/R Veh | I/R α-LA |
|  | 24.33±2.17(4) | 24.99±1.39(4) | 14.58±1.82(4)* | 24.96±1.85(4)^##^ |
| 7c | **RBPMS^+^ cells (n) day-7** | | | |
|  | CTL Veh | CTL α-LA | I/R Veh | I/R α-LA |
|  | 26.43±1.56(4) | 25.63±2.08(4) | 17.92±1.12(4)** | 24.37±0.63(4)^#^ |
| 7d | **4-HNE (mean grey value) in GCL** | | | |
|  | CTL (*Trpa1^+/+^*) | CTL (*Trpa1^-/^*^-^) | I/R (*Trpa1^+/+^*) | I/R (*Trpa1^-/-^*) |
|  | 1.86±0.11(4) | 2.09±0.27(4) | 5.33±0.66(4)*** | 1.99±0.30(4)^###^ |
|  | **4-HNE (mean grey value) in INL** | | | |
|  | CTL (*Trpa1^+/+^*) | CTL (*Trpa1^-/^*^-^) | I/R (*Trpa1^+/+^*) | I/R (*Trpa1^-/-^*) |
|  | 1.79±0.09(4) | 1.84±0.14(4) | 4.91±0.55(4)*** | 1.97±0.27(4)^###^ |
| 7e | **4-HNE (mean grey value) in GCL day-2** | | | |
|  | CTL Veh | CTL A96 | I/R Veh | I/R A96 |
|  | 18.21±1.17(4) | 20.08±0.87(4) | 23.68±1.30(4)* | 19.26±0.76(4)^#^ |
|  | **4-HNE (mean grey value) in INL day-2** | | | |
|  | CTL Veh | CTL A96 | I/R Veh | I/R A96 |
|  | 15.30±1.18(4) | 16.44±0.61(4) | 20.67±0.98(4)** | 17.06±0.13(4)^#^ |
| 7f | **4-HNE (mean grey value) in GCL day-2** | | | |
|  | CTL Veh | CTL HC03 | I/R Veh | I/R HC03 |
|  | 10.20±0.54(4) | 9.78±0.11(4) | 18.05±0.98(4)*** | 12.15±0.44(4)^###^ |
|  | **4-HNE (mean grey value) in INL day-2** | | | |
|  | CTL Veh | CTL HC03 | I/R Veh | I/R HC03 |
|  | 10.58±0.58(4) | 10.10±0.25(4) | 19.91±0.70(4)*** | 12.63±0.53(4)^###^ |
| 7g | **4-HNE (mean grey value) in GCL day-7** | | | |
|  | CTL Veh | CTL HC03 | I/R Veh | I/R HC03 |
|  | 13.02±1.33(4) | 12.23±0.50(4) | 21.63±2.16(4)** | 14.26±0.86(4)^##^ |
|  | **4-HNE (mean grey value) in INL day-7** | | | |
|  | CTL Veh | CTL HC03 | I/R Veh | I/R HC03 |
|  | 13.85±1.78(4) | 13.02±0.66(4) | 21.47±1.45(4)** | 14.65±1.04(4)^#^ |
| *P<0.05, **P<0.01, ***P<0.001 *vs*. CTL (*Trpa1^+/+^*) or CTL Veh  ^#^P<0.05, ^##^P<0.01, ^###^P<0.001 *vs*. I/R (*Trpa1^+/+^*) or I/R Veh | | | | |
| **Supplemental Fig 8** | | | | |
| 8a | **F4/80^+^ cells (n)** | | | |
|  | CTL (*Trpa1^+/+^*) | CTL (*Trpa1^-/^*^-^) | I/R (*Trpa1^+/+^*) | I/R (*Trpa1^-/-^*) |
|  | 11.88±0.76(5) | 11.88±1.02(5) | 20.75±1.23(5)* | 14.48±1.24(5)^#^ |
| 8b | **F4/80^+^ cells (n)** | | | |
|  | CTL Veh | CTL A96 | I/R Veh | I/R A96 |
|  | 12.00±0.56(4) | 13.13±0.59(4) | 17.71±0.94(4)*** | 12.69±0.38(4)^###^ |
| 8c | **F4/80^+^ cells (n) day-2** | | | |
|  | CTL Veh | CTL HC03 | I/R Veh | I/R HC03 |
|  | 14.08±0.32(4) | 13.60±0.75(4) | 17.23±0.22(4)* | 12.72±1.16(4)^#^ |
| 8d | **F4/80^+^ cells (n) day-7** | | | |
|  | CTL Veh | CTL HC03 | I/R Veh | I/R HC03 |
|  | 10.13±0.64(4) | 10.45±1.10(4) | 15.22±1.45(4)* | 10.72±0.55(4)^#^ |
| 8e | **F4/80^+^ cells (n) day-2** | | | |
|  | CTL PBS | CTL LCL | I/R PBS | I/R LCL |
|  | 7.60±0.30(4) | 6.36±1.08(4) | 16.40±0.30(4)*** | 6.60±0.46(4) |
| 8f | **Retinal thickness (µm) day-2** | | | |
|  | CTL PBS | CTL LCL | I/R PBS | I/R LCL |
|  | 87.65±1.43(4) | 78.38±7.28(4) | 61.95±1.41(4)** | 55.02±3.19(4) |
| 8g | **4-HNE (mean grey value) in GCL day-2** | | | |
|  | CTL PBS | CTL LCL | I/R PBS | I/R LCL |
|  | 10.99±0.36(4) | 10.82±0.13(4) | 13.57±0.30(4)** | 13.37±0.35(4) |
|  | **4-HNE (mean grey value) in INL day-2** | | | |
|  | CTL PBS | CTL LCL | I/R PBS | I/R LCL |
|  | 11.11±0.34(4) | 11.57±0.33(4) | 14.69±0.33(4)** | 14.52±0.72(4) |
| 8h | **Active caspase-3 (mean grey value) day-2** | | | |
|  | CTL PBS | CTL LCL | I/R PBS | I/R LCL |
|  | 4.63±0.14(4) | 4.99±0.23(4) | 8.05±0.80(4)* | 10.06±0.96(4) |
| *P<0.05, **P<0.001, ***P<0.001 *vs.* CTL (*Trpa1^+/+^*) or CTL Veh  ^#^P<0.05, ###P<0.001 *vs.* I/R (*Trpa1^+/+^*) or I/R Veh | | | | |

CTL, control; Veh, vehicle; I/R, ischemia/reperfusion; HC03, HC-030031; A96, A-967079; LCL, liposome-encapsulated clodronate; α-LA, α-lipoic acid; Indo, indomethacin; GCL, ganglion cell layer; INL, inner nuclear layer; RBPMS, RNA binding protein with multiple splicing; GAD67, glutamic acid decarboxylase; GS, glutamine synthetase; 4-HNE, 4-hydroxynonenal. Two-way ANOVA followed by post-hoc comparisons using the Bonferroni correction
