# Supplementary figures and images for: TRPA1 mediates damage of the retina induced by ischemia and reperfusion in mice
Source: Cell Death Dis. 2020 Aug 15;11(8):633. doi: 10.1038/s41419-020-02863-6 (PMC7429961; doi:10.1038/s41419-020-02863-6)

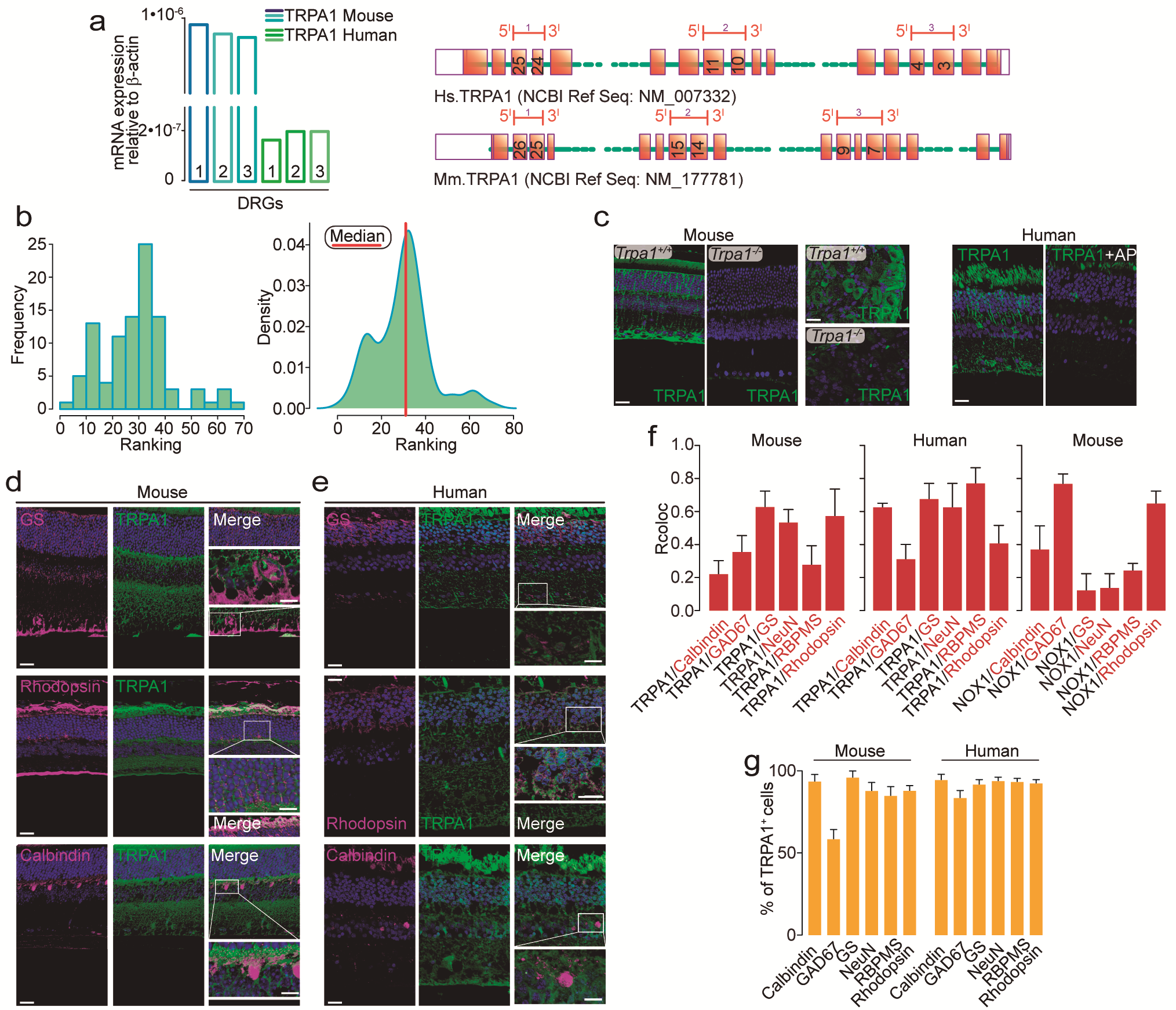

Supplement: Supplementary file 3 — Supplemental Figure 1 [file 41419_2020_2863_MOESM3_ESM.tif]

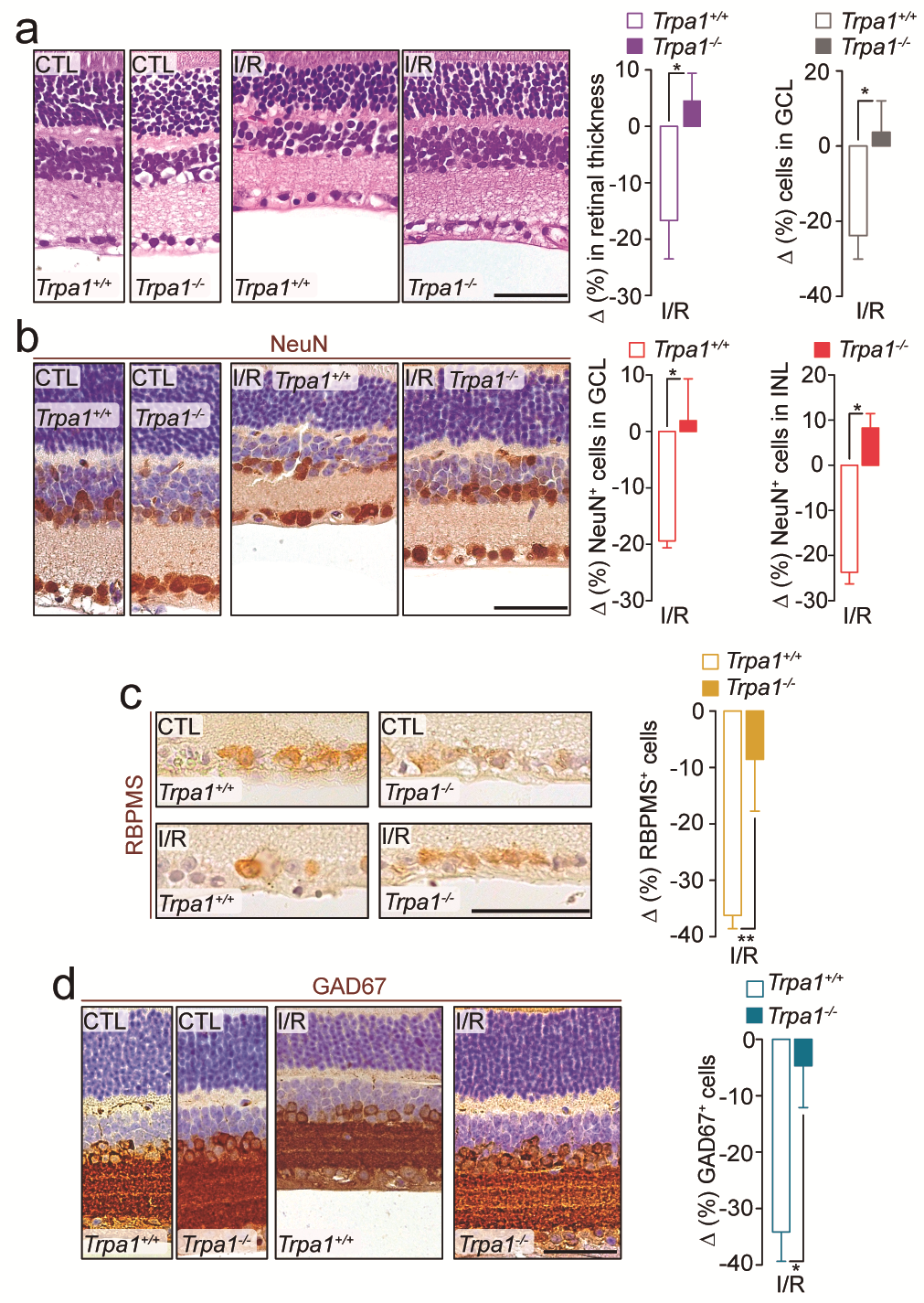

Supplement: Supplementary file 4 — Supplemental Figure2 [file 41419_2020_2863_MOESM4_ESM.tif]

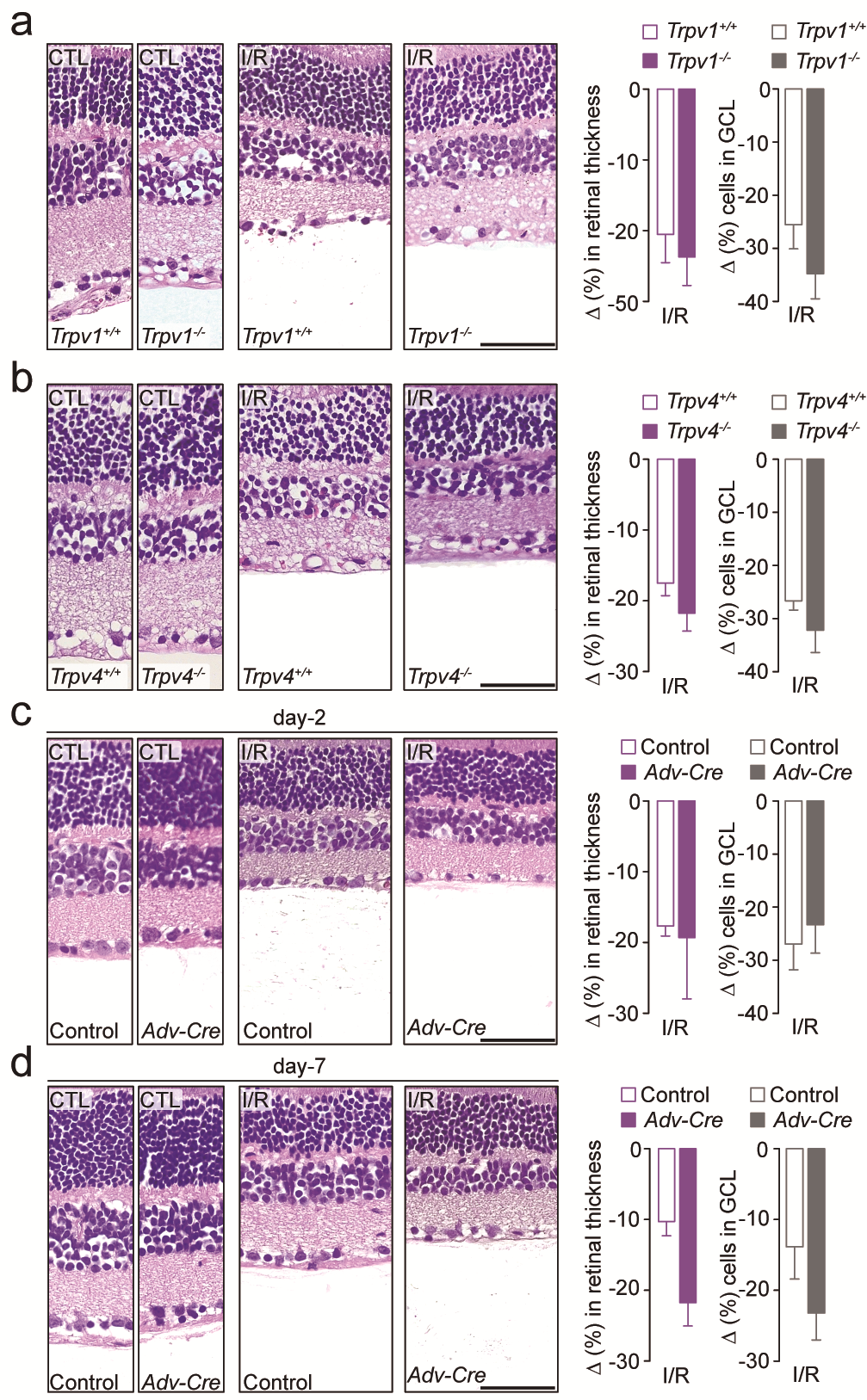

Supplement: Supplementary file 5 — Supplemental Figure 3 [file 41419_2020_2863_MOESM5_ESM.tif]

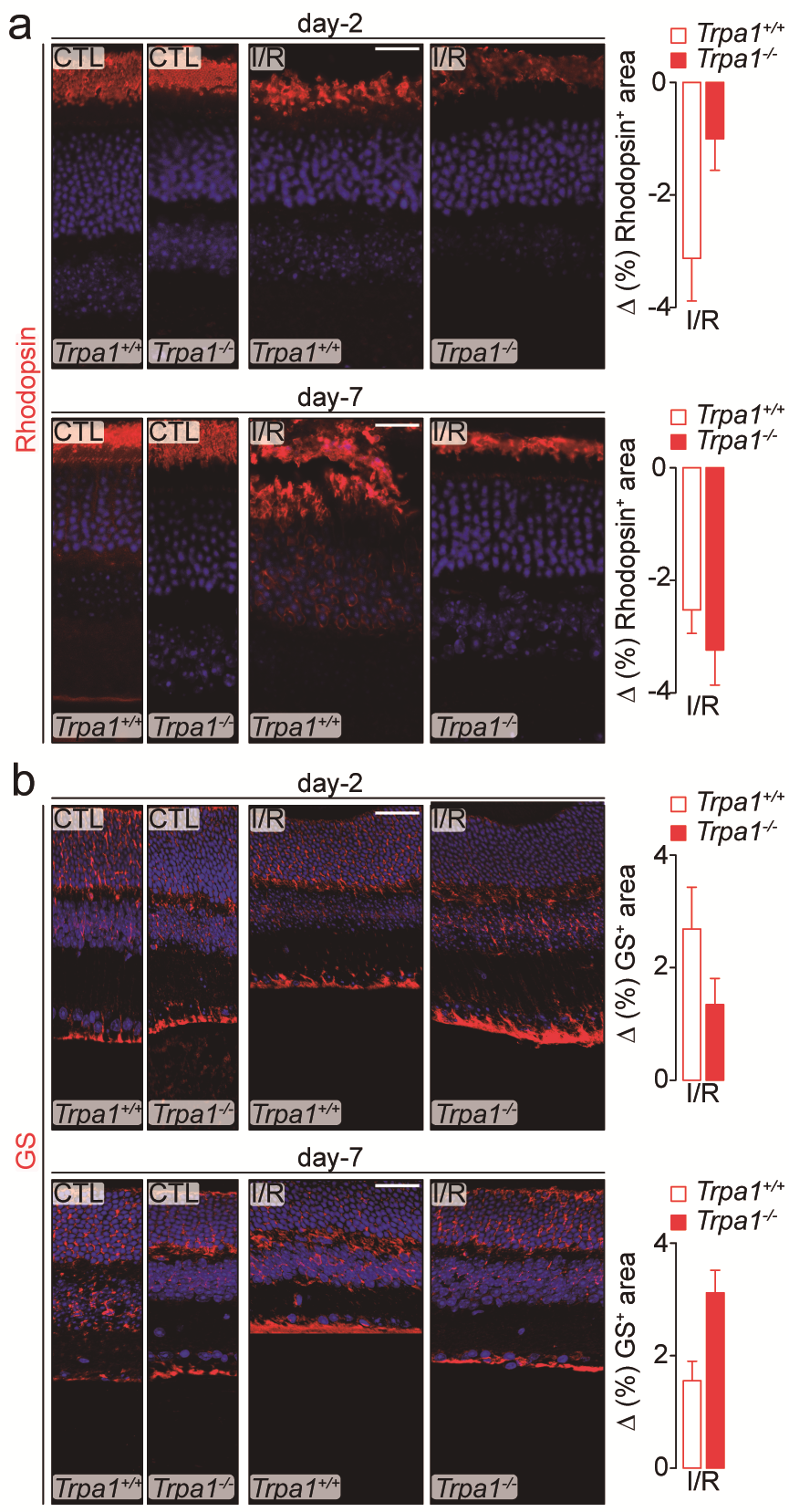

Supplement: Supplementary file 6 — Supplemental Figure 4 [file 41419_2020_2863_MOESM6_ESM.tif]

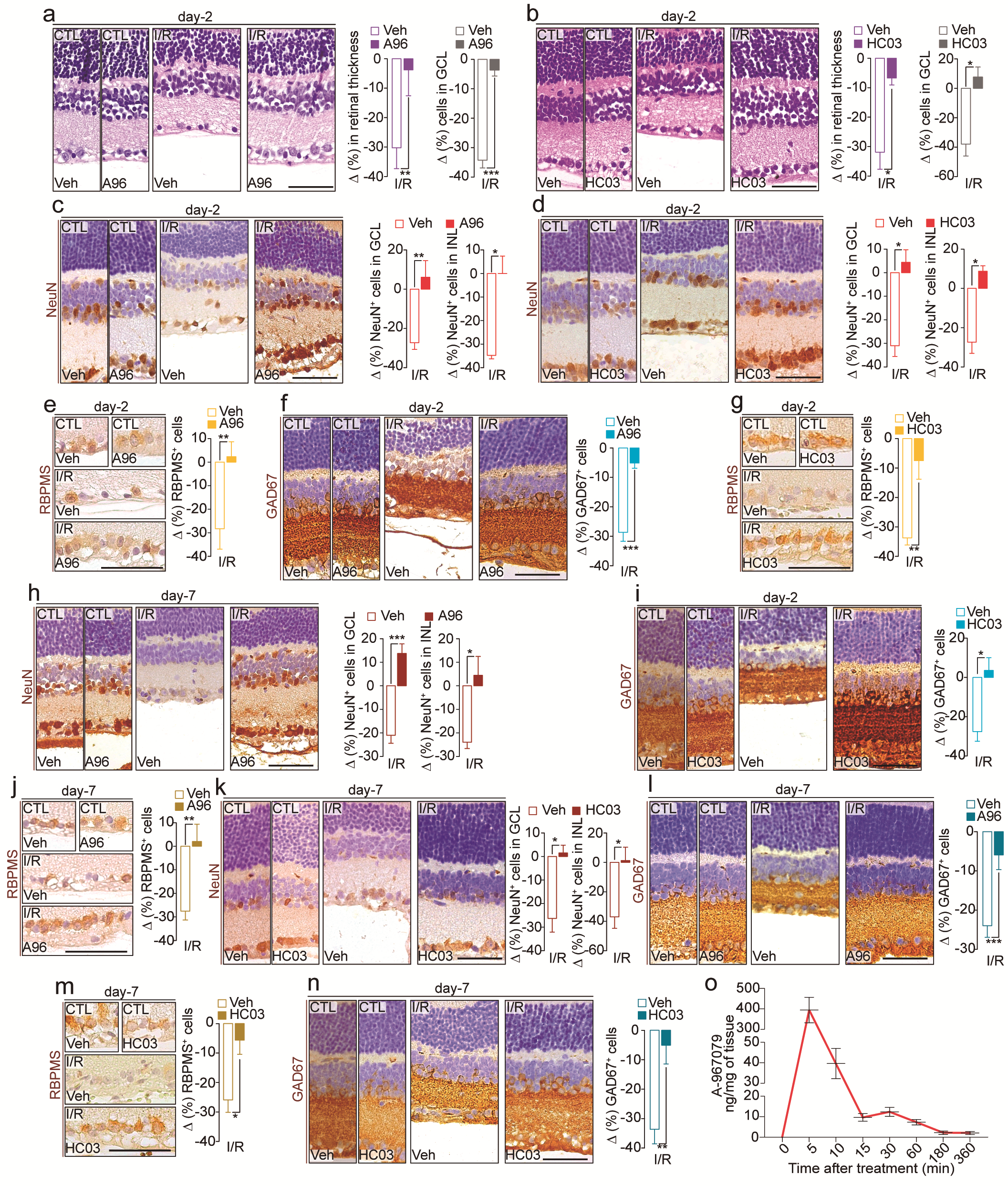

Supplement: Supplementary file 7 — Supplemental Figure 5 [file 41419_2020_2863_MOESM7_ESM.tif]

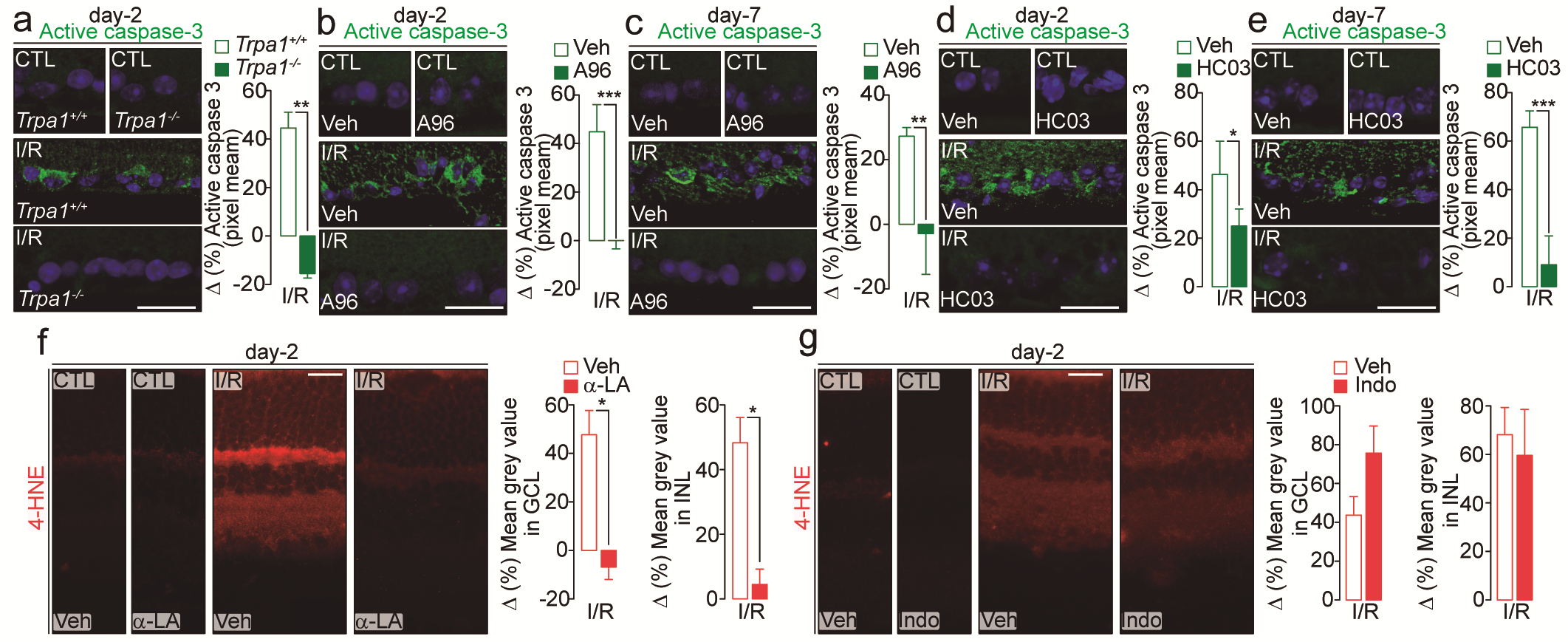

Supplement: Supplementary file 8 — Supplemental Figure 6 [file 41419_2020_2863_MOESM8_ESM.tif]

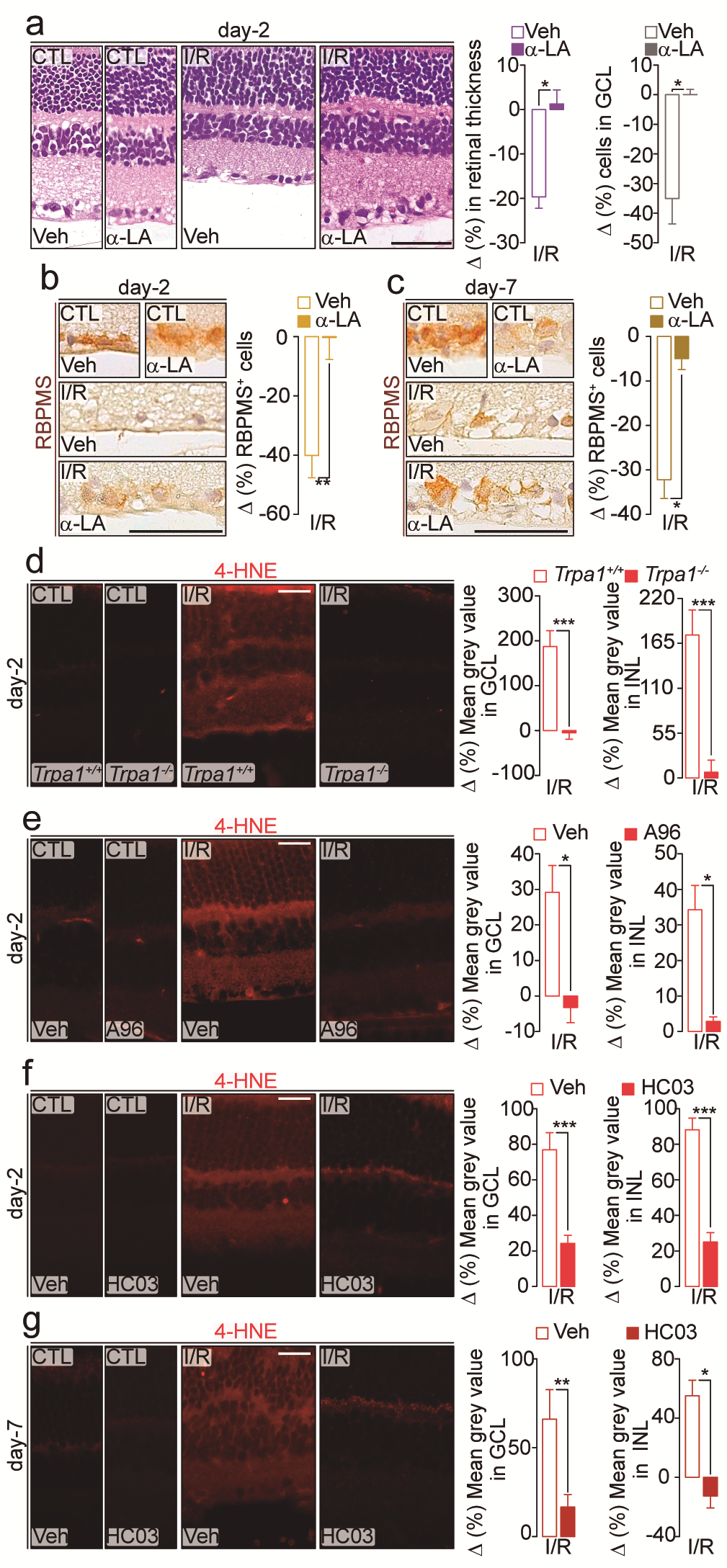

Supplement: Supplementary file 9 — Supplemental Figure 7 [file 41419_2020_2863_MOESM9_ESM.tif]

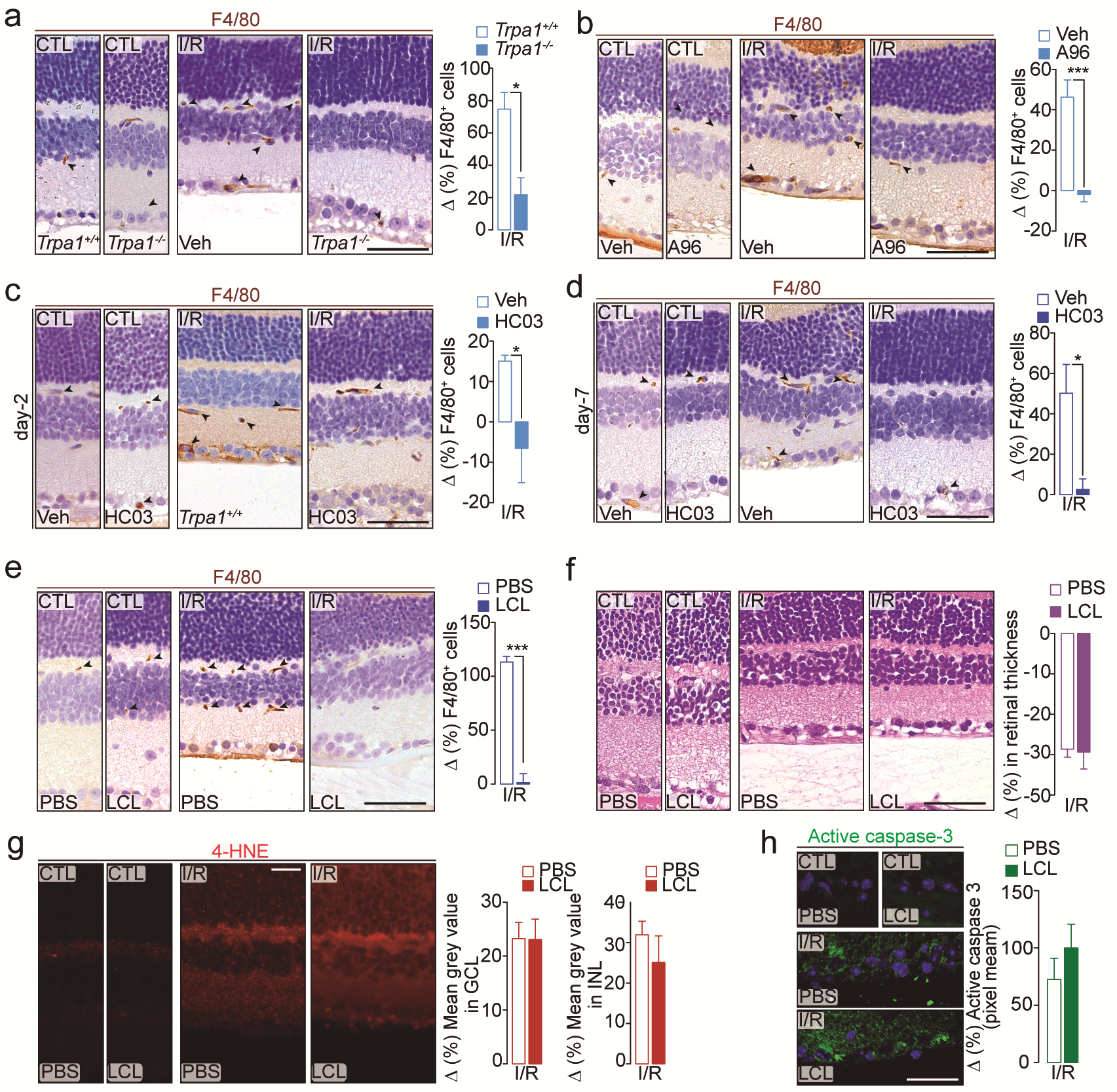

Supplement: Supplementary file 10 — Supplemental Figure 8 [file 41419_2020_2863_MOESM10_ESM.tif]

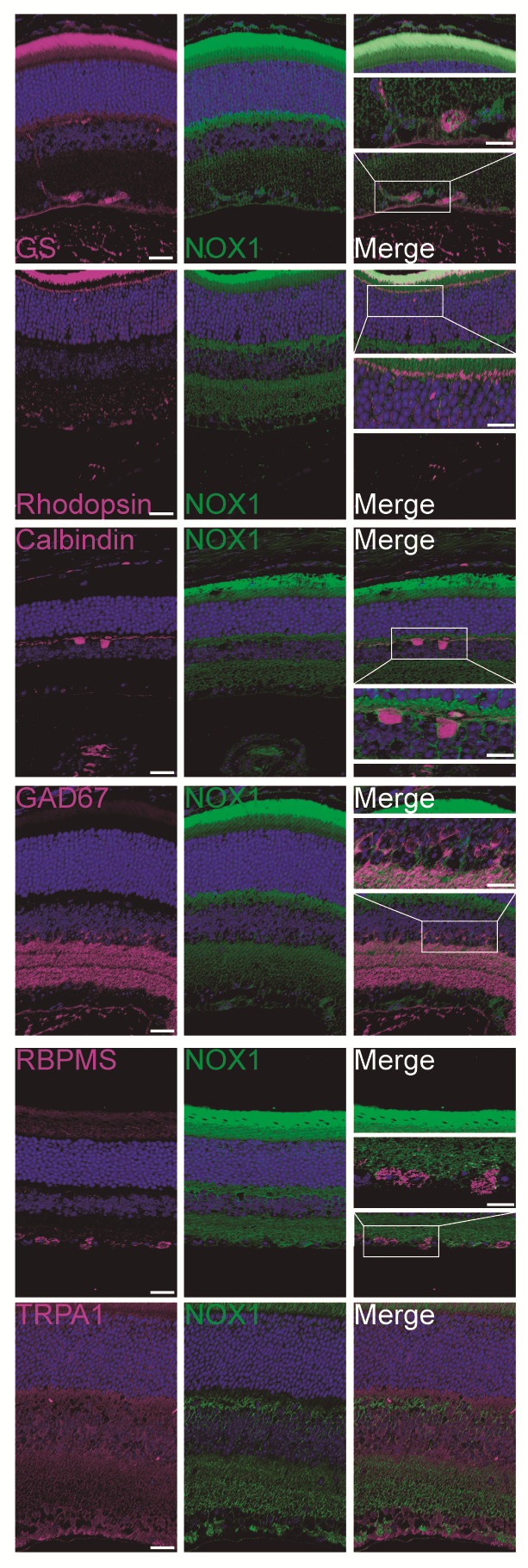

Supplement: Supplementary file 11 — Supplemental Figure 9 [file 41419_2020_2863_MOESM11_ESM.tif]
